# Spatial variation in food web structure in a recovering marine ecosystem

**DOI:** 10.1371/journal.pone.0268440

**Published:** 2022-05-20

**Authors:** Kyle J. Krumsick, Jonathan A. D. Fisher

**Affiliations:** Centre for Fisheries Ecosystems Research, Fisheries and Marine Institute of Memorial University of Newfoundland, St. John’s, Newfoundland, Canada; MARE – Marine and Environmental Sciences Centre, PORTUGAL

## Abstract

Spatial heterogeneity in food web structure and interactions may reconcile spatial variation in population and community dynamics in large marine ecosystems. In order to assess food web contributions to the different community recovery dynamics along the Newfoundland and Labrador shelf ecosystem, we quantified species interactions using stable isotope mixing models and food web metrics within three sub-regions. Representative samples of each species caught in trawls and plankton tows were analyzed for stomach contents and stable isotope ratios (δ^15^N and δ^13^C) to parameterize isotope mixing models. Regional variation, highlighted by the diets of three economically important species, was observed such that the southern region demonstrated a variety of trophic pathways of nutrient flow into the higher food web while the diets of fish in the northern regions were typically dominated by one or two pathways via dominant prey species, specifically shrimp (*Pandalus* sp.) and hyperiids. Food web metrics indicated that the low-diversity northern regions had higher connectance and shorter food chain lengths. This observed regional variation contributes to our understanding of the role of specific forage species to the ecosystem which is an essential contribution towards ecosystem-based management decisions.

## Introduction

Quantifying spatial variation in ecosystem processes can be key to understanding the functioning and dynamics within large marine ecosystems [1, 2]. The collapse of demersal fish stocks off Newfoundland and Labrador (northwest Atlantic Ocean) brought about ecosystem changes by reducing the influence of dominant top predators [3]. One impact of this change was interactions among species through predation and indirect effects [4]. Now, more than two decades after stock collapses and initial fisheries moratoria, the ecosystem has not fully recovered [5, 6]. Factors hypothesized to influence population trajectories include continued fishing [7], depensatory growth [8], climate change [9], and life history changes [10]. However, Newfoundland and Labrador marine ecosystems also exhibit spatial heterogeneity in their structure and function with northern communities displaying lower fish species diversity and diet diversity [6, 11], and lower recovery of marine fish community size-structure [6]. In an ecosystem context, it is important to quantify the extent to which spatial variability in diets on relevant timescales may also contribute to differing population and community trajectories.

Historically the Newfoundland and Labrador region had been managed focusing on single species, contributing to the collapse of groundfish stocks in the regions in the early 1990s [12–15]. It has since become apparent that managing a stock in isolation is not sufficient and that managing the ecosystem with complex interactions with multiple species would help to facilitate future sustainable fishing practices [16–19]. Given interest in the application of ecosystem-based analyses to fisheries management, understanding food-web interactions and their contribution to species and community dynamics has become essential [16, 20, 21]. These trophic interactions represent a key factor which regulates fish populations [22–25]. Management of stocks requires consideration of trophic interactions which may confound indicators used to assess effectiveness of management measures and can influence management decisions when multiple exploited species exist, either through assessing the trade-offs of exploiting each species in the case of direct trophic interactions or determining the ecosystem-level impacts of fishing pressures [26–28]. Quantifying trophic interactions has relied on various methodologies and tools including stomach content analysis [29–31], fatty acid analyses [31–34], pyrosequencing of prey DNA from stomach contents or faeces [35–37], and stable isotope analyses [38].

Stable isotope analyses constitutes the primary technique that has been used to supplement stomach contents analyses for assessing energy flow through an ecosystem over longer periods [39, 40]. Stable isotopes in ecology provide information on the origins and consequent assimilation of organic matter which provides insights into food web [41–44]. Together these two isotope values can be used to determine isotopic niches which are thought to be proxies of trophic niches [45–47]. Importantly, with additional inputs of prey isotopic values, fractionation coefficient estimates, and prey elemental composition, the approximate proportions of the predator diet can be estimated using isotope mixing models [48, 49]. The isotope mixing model used herein is designed to quantify the relative contributions of the pelagic/benthic food chains, determine the relative trophic position of members of the community and to quantify the trophic relationships between organisms. The results of this isotope mixing model will allow for reconstruction of the marine food web to allow for assessment of the direct and indirect interactions between organisms as well as the important species for the passage of energy through the food web.

Three of the dominant, economically important groundfish species found along the Newfoundland and Labrador shelves are Atlantic cod (*Gadus morhua*), redfish (*Sebastes* sp.) and Greenland halibut (*Reinhardtius hippoglossoides*). Between 2013 and 2017 these three species represented 78.1% of the landed value of groundfish species in the Newfoundland-Labrador region [50]. Given their relative importance to the fisheries in this region, several historical diet studies have been conducted using stomach contents analyses. The demersal Atlantic cod are thought to be primarily generalist feeders but do show preferences towards high-lipid forage fish such as capelin [e.g. 11, 51–53]. The largely pelagic redfish has been reported to feed primarily on pelagic invertebrates such as hyperiid amphipods, copepods, euphausiids, and northern shrimp [e.g. 54–57]. The Greenland halibut is primarily a demersal fish but is thought to be an active mid-water predator. As such, their diet consists of a wide variety of pelagic and demersal prey, particularly capelin, shrimp, squid, and zooplankton [e.g. 58–61]. All of these diets have been reported to have changed along with the large-scale ecosystem changes that were observed in this region in the early 1990s [57, 61, 62].

Given the utility of stable isotope analyses to contribute new information on the structure of food webs and trophic dynamics among ecosystems, our objectives are threefold: (a) to construct simplified marine food webs based on results from stable isotope mixing models supplemented by information from stomach contents data for three regions within the northeast coast of Newfoundland and Labrador and then compare food web metrics among regions; (b) to analyze in greater detail spatial variation in the diets of the three abundant and economically important species: Atlantic cod, redfish and Greenland halibut, and; (c) to discuss the isotope mixing model results in the context of recently reported spatial variation in marine fish ontogenetic niche overlap and size-spectrum recovery among regions. Together these results contribute new information towards ecosystem-based decisions by quantifying the relative importance of specific forage species to these communities.

## Materials and methods

### Study area

**Fig 1:**
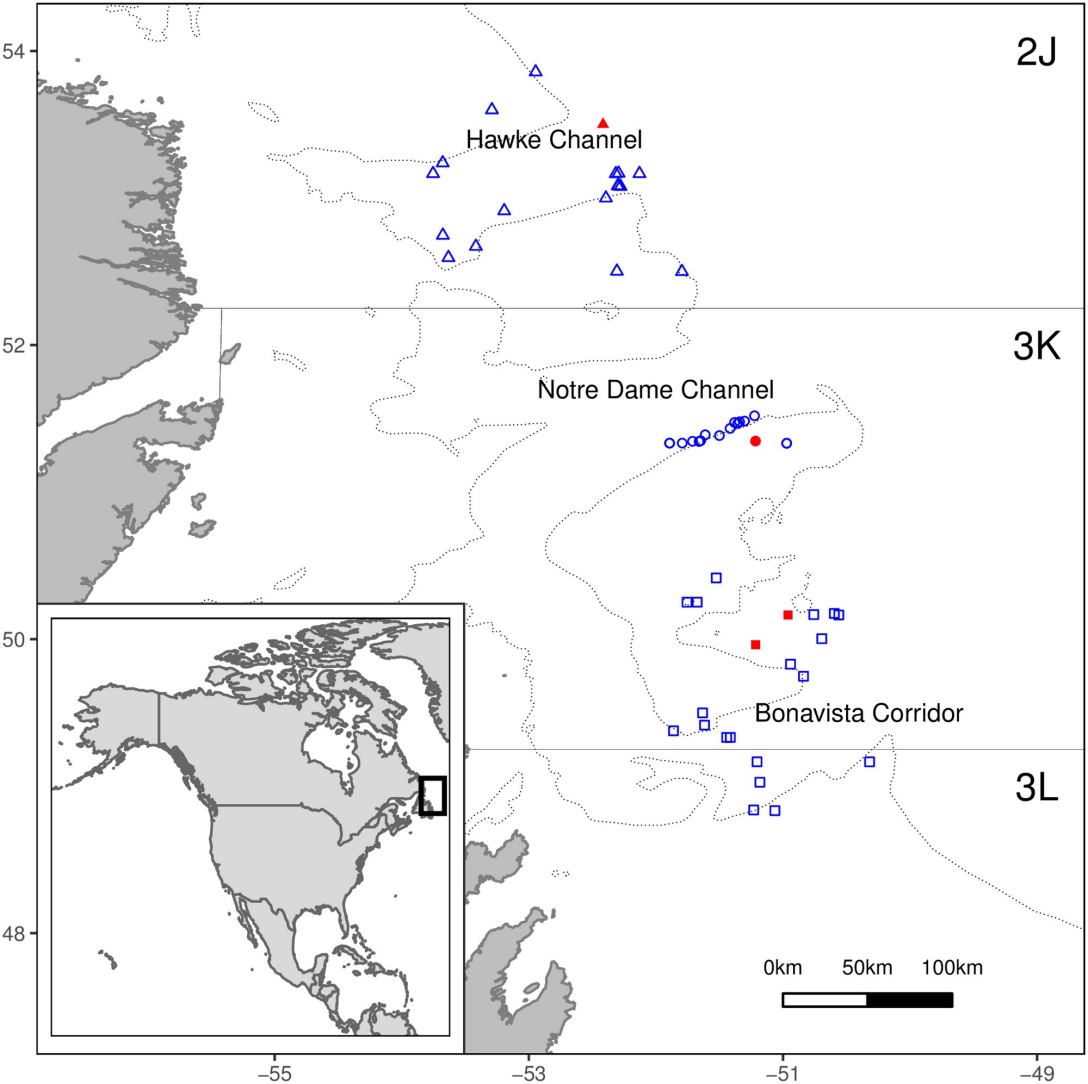
Map of Newfoundland and southern Labrador with sampling locations. The trawl locations are indicated with open blue symbols and plankton tow locations with closed red symbols for the Hawke Channel (HC, triangles), Notre Dame Channel (NDC, circles), and Bonavista Corridor (BC, squares). The inset map outlines the study domain in eastern Canada. The relevant NAFO subdivisions 2J, 3K, and 3L boundaries are also indicated. Dashed lines represent 300 m depth contours. The bathymetry map is reproduced from GEBCO world map 2020 (www.gebco.net) and NAFO subdivisions reproduced from NAFO (www.nafo.int).

Sampling was undertaken within marine research surveys conducted by the Center for Fisheries Ecosystems Research (CFER) aboard the *RV Celtic Explorer* in May 2015 on the offshore shelves from southern Labrador and eastern Newfoundland, corresponding to Northwest Atlantic Fishery Organization (NAFO) subdivisions 2J, 3K, and 3L (Fig 1). These subdivisions together represent the management unit for the ‘Northern’ Atlantic cod stock [63–65]. Three major channels or corridors within this region had previously been identified as important onshore-offshore cod migration pathways: Hawke Channel, Notre Dame Channel, and the Bonavista Corridor [66]. The following analyses were conducted within each channel separately in order to quantify spatial variation.

### Sample collection

Animal research was provided written consent by the Animal Care Committee of Memorial University of Newfoundland and Labrador. Field research and sample collection was conducted under a Fisheries and Ocean Canada experimental license (NL-2927-15). The collection of samples used for this study was reviewed and approved by the Memorial University Animal Care Committee under the guidelines set by the Canadian Council of Animal Care. In order to sample zooplankton, bongo nets (33 μm mesh and 60 cm diameter) were deployed obliquely within the surface layer for 10 minutes and at a speed of 2 knots. Fish and invertebrate samples were collected using a combination of Campelen 1800 and mid-water trawls deployed at depths ranging from 244 m to 471 m [47]. All trawl sets were deployed between the hours of 7 am and 10 pm. All fish caught in the trawl sets were sorted by species and standard lengths recorded. In cases where a species was particularly abundant, subsampling for length was initiated involving 100 randomly sampled individuals. Individuals were selected for dissection with efforts to provide representation from the observed size ranges for each species. For the majority of species, only nine specimens were collected within each region with the exception of species protected by the Species at Risk Act (Table 1). From most fish, a transverse sample of dorsal muscle tissue directly posterior to the head was collected at-sea, placed in a 1.5 ml centrifuge vial and frozen at -20 ºC for stable isotope analyses. Stomachs were also collected at sea and preserved frozen. Stomachs from fish that showed signs of regurgitation or stomach eversion were noted but not collected due to the potential of biasing stomach content results. The remaining fish with small, difficult to sample stomachs were individually labelled, bagged whole, and preserved frozen for dissection in the laboratory for their muscle tissue and stomachs.

**Table 1:** Species included in this study, their species abbreviation, and the group category to which they were assigned.

Species Number	Scientific Name	Common Name	Classification Category
**1**	*Gadus morhua*	Atlantic Cod	Demersal Fish
**10**	*Clupea harrengus harrengus*	Atlantic Herring	Pelagic Fish
**11**	*Aspidophoroides monopterygius*	Alligatorfish	Demersal Fish
**25**	Gammaridae	Gammarid Amphipod	Pelagic Invert
**2**	*Hippoglossoides platessoides*	American Plaice	Demersal Fish
**12**	*Boreogadus saida*	Arctic Cod	Pelagic Fish
**26**	Chaetognatha	Arrow Worm	Pelagic Invert
**13**	*Notolepis rissoi*	White Barracudina	Pelagic Fish
**22**	*Benthosema glaciale*	Glacier Lanternfish	Pelagic Fish
**27**	*Gorgonocephalus arcticus*	Basket Star	Benthic Invert
**50**	-	Benthic Plants	Benthic Plant
**28**	*Ophiopholus aculeata*	Brittle Star	Benthic Invert
**29**	Bivalva	Bivalve	Benthic Invert
**3**	*Mallotus villosus*	Capelin	Pelagic Fish
**4**	*Lycodes vahlii*	Checkered Eelpout	Demersal Fish
**30**	Copepoda	Copepod	Pelagic Invert
**31**	Euphausiacea	Euphausiid	Pelagic Invert
**32**	*Buccinum undatum*	Whelk	Benthic Invert
**5**	*Reinhardtius hippoglossoides*	Greenland Halibut / Turbot	Pelagic Fish
**23**	*Macruronus novaezelandiae*	Blue Hake	Demersal Fish
**33**	Holothuroidea	Sea Cucumber	Benthic Invert
**14**	*Artediellus atlanticus*	Hookear Sculpin	Demersal Fish
**34**	Hyperiidea	Hyperiid Amphipod	Pelagic Invert
**46**	Isopoda	Isopod	Benthic Invert
**15**	*Urophycis chesteri*	Longfin Hake	Demersal Fish
**35**	Mysidae	Mysid	Pelagic Invert
**6**	*Nezumia bairdi*	Marlinspike Grenadier	Demersal Fish
**16**	*Triglops murrayi*	Moustache Sculpin	Demersal Fish
**17**	*Notoscopelus* sp.	Krøyer’s Lanternfish	Pelagic Fish
**47**	Nudibranchia	Nudibranch	Benthic Invert
**49**	Ostracoda	Ostracod	Pelagic Invert
**36**	Polychaeta	Polychaete	Benthic Invert
**51**	-	Pelagic Algae	Pelagic Plant
**18**	*Agonus decagonus*	Atlantic Poacher	Demersal Fish
**37**	Pantopoda	Pycnogonid	Benthic Invert
**24**	*Macrourus berglax*	Roughhead Grenadier	Demersal Fish
**7**	*Sebastes* sp.	Redfish	Pelagic Fish
**38**	Actiniaria	Sea Anemone	Benthic Invert
**19**	*Lumpenus lumpretaeformis*	Snakeblenny	Demersal Fish
**40**	*Chionocetes opilio*	Snow Crab	Benthic Invert
**41**	Asteroidea	Sea Star	Benthic Invert
**39**	*Sabinea sarsii*	Shrimp	Benthic Invert
**42**	*Pandalus* sp.	Shrimp	Benthic Invert
**43**	Sipuncula	Sipunculid	Benthic Invert
**44**	Decapodiformes	Squid	Pelagic Invert
**20**	*Raja senta*	Smooth Skate	Demersal Fish
**48**	*Strongylocentrotus droebachiensis*	Sea Urchin	Benthic Invert
**44**	*Hyas* sp.	Toad Crab	Benthic Invert
**21**	*Gaidropsarus ensis*	Three-beard Rockling	Demersal Fish
**8**	*Raja radiata*	Thorny Skate	Demersal Fish
**9**	*Glyptocephalus cynoglossus*	Witch Flounder	Demersal Fish

While more species were caught and analyzed for their isotopic signatures (Table 1), nine species represented the most abundant fish species: American plaice (*Hippoglossoides platessoides*), Atlantic cod (*Gadus morhua*), Atlantic herring (*Clupea harengus harengus*), capelin (*Mallotus villosus*), checker eelpout (*Lycodes vahlii*), Greenland halibut (*Reinhardtius hippoglossoides*), lanternfish (N*otoscopelus* sp.), redfish (*Sebastes* sp.), and thorny skate (*Amblyraja radiata*). Based on the species’ observed length distributions, sampled individuals were classified as small, medium, or large, by dividing the observed range of sizes into three length categories of equal width (Table 2). Given the more extensive analyses of these species, sampling efforts were increased to include seven individuals within each size category. These categories are recognized to be arbitrary, but as the exact timing of potential ontogenetic shifts was unknown, this division accounted for variation across the range of observed sizes. Of these, Atlantic cod, Greenland halibut, and redfish were selected to portray regional variability given their abundances and economic importance.

**Table 2:** Definition of small, medium and large size categories for the most abundant nine fish species. Size category definitions were consistent across regions.

Species	Small size range (cm)	Medium size range (cm)	Large size range (cm)
American Plaice	7.0–22.6	22.7–38.3	38.4–54.0
Atlantic Cod	13.0–45.9	46.0–80.0	80.1–113.0
Atlantic Herring	27.1–30.9	31.0–34.7	34.8–38.5
Capelin	11.0–13.6	13.7–16.2	16.3–18.8
Checker Eelpout	8.0–20.9	21.0–33.9	34.0–47.0
Greenland Halibut	10.0–27.4	27.5–45.0	45.1–62.5
Lanternfish	12.9–14.5	14.6–15.6	15.7–17.4
Redfish	4.0–18.6	18.7–33.2	33.3–48.0
Thorny Skate	10.2–33.9	34.0–58.3	58.4–80.0

In addition to the fish samples, a variety of invertebrates were collected (Table 1). Invertebrates were also sorted by species and measurements such as carapace width in crabs and carapace length in shrimp were obtained for up to 100 randomly sampled individuals. A sample of up to twenty-one of each invertebrate per region were frozen whole, except for large snow crab, each of which was sampled by removing one leg. Zooplankton were collected from each plankton tow passed through a 140-micron sieve and were preserved frozen for further taxonomical identification in the laboratory.

### Stomach content analysis

Stomach samples were analyzed using a dissecting microscope and contents were identified to the lowest feasible taxonomic level. Individual weights and numbers of each prey taxa were quantified. From these measurements, in combination with their frequency of occurrence, the index of relative importance (IRI) was calculated for each prey taxa as follows:

$$IRI=\left(\%N+\%B\right)\;\left(FO\right)$$ (1)

Where %N is the percent contribution of a given taxon to stomach content by numbers, %B is its percent contribution by weight, and FO is the frequency of occurrence, defined as the number of stomachs in which the prey taxon was detected over the total number of stomachs [67]. In combining these three measures into a single index, it should free the analysis from any biases associated with each of the measures independently. The percent IRI is presented as a percentage of the summation of the IRIs of all prey observed and were calculated for each predator species and region combination.

### Stable isotope analysis

Muscle tissue samples were oven dried at 75°C for 48 hours and homogenized using an amalgamator. The homogenized samples were weighed, packaged in an airtight container with desiccant packages and shipped to the Cornell University Stable Isotope Laboratory (Ithaca, NY, USA) for analysis. Approximately 1 mg of sample was placed into 7×7 mm tin capsules, then flash combusted using a Carlo-Erba NC2500 elemental analyzer coupled on-line to a Finnigan MAT Delta Plus mass spectrometer for analyses of the resulting carbon dioxide and nitrogen gases.

The stable nitrogen isotope signature (*δ*^15^*N*) typically becomes enriched by approximately 3 ‰ for fish species with each consumption, allowing for approximation of trophic level [68–70]. The stable carbon isotope signature (*δ*^13^*C*) provides an indication of the initial carbon source (pelagic or benthic/detrital in origin) and enriches at typically less than 1 ‰ with fractionation frequently considered negligible [71–73]. Nitrogen and carbon ratios were expressed in delta (*δ*) notation, being the parts per thousand deviation from the standard material, Pee Dee belemnite limestone for carbon and atmospheric nitrogen for nitrogen, as follows:

$$\delta^{15}N\;or\;\delta^{13}C=\left(\left(\frac{\_R_{sample}\_}{R_{standard}}\right)-1\right)\times 1000$$ (2)

R=C13/C12orN15/N14 (3)

Lipids were not removed to avoid the potential influence of derived products on isotopic signatures [74–76]. Therefore, following analysis, the *δ*^13^ C values were normalized for lipid bias as recommended by Ricklefs & Travis [77] and Post *et al*. [78], as follows:

$$\delta^{13}C_{normalized}=\delta^{13}C_{untreated}-3.32+0.99\times C:\;N$$ (4)

As the majority of fish samples possessed a carbon to nitrogen concentration ratio close to 3.3, as would be expected for muscle tissue of most marine fish [77], minimal modification between the normalized and untreated carbon values was observed for the majority of analyzed fish. This adjustment was only particularly relevant for lipid rich fish, such as capelin, lanternfish and Greenland halibut, where the carbon concentration becomes increased relative to the nitrogen.

### Stable isotope mixing model

Although stomach contents provide detailed information on the diet composition of species, it represents the diet on the scale of hours to days [79–81]. In order to assess longer-term prey consumption and food web connections on the scale of months, we analyzed the diets of every caught predatory fish species (a total of 25 species) using Bayesian concentration-weighted stable isotope mixing models. Eight of these 25 species were also found to represent a at least three size categories as defined by Table 2, for a total of 41 separate models. These models approximate the contributions of different prey while assessing their locations in isotope space relative to adjusted predator values. These techniques are partly informed by priors, in our case stomach contents data. These priors were separated by region where possible, but in cases where a given region was represented by three or fewer stomachs combined stomach data for all regions was input as the prior. Species or functional groups were further divided into four categories based on descriptions provided by Sherwood & Rose [82] and Scott & Scott [83] (Table 1): benthic invertebrates, pelagic invertebrates, demersal fish, and pelagic fish. Some species, such as copepod species, were not found to differ in their isotopic signature and therefore were pooled together as a single functional group.

A fractionation coefficient, or discrimination factor, is the change in the isotopic signature from prey to predator that occurs due to partitioning upon consumption and assimilation of respective elements. Determining the exact fractionation coefficients between the predator and each individual food source is often recommended for each element [84]. Such determination of prey-specific fractionation coefficients, however, was not possible in the present study. Historically a nitrogen fractionation of 3.4 has been used in ecological studies of fish populations and 0 for carbon, as if fractionation was assumed to be negligible [69, 71, 85]. However, these carbon estimates may have been underestimated and nitrogen overestimated [86]. As a result, two approaches were taken to estimate these discrimination factors. The coefficient was first estimated from the combination of stomach content analysis and the associated isotopic values of prey per Sherwood and Rose [82]. The bounds of values were 1.4–4.4 for nitrogen and -0.5–2 for carbon, as determined by biologically feasible fractionation coefficients [38, 73, 84, 86–90]. For estimated coefficients outside these bounds, the fractionation was estimated to be 3.4 for nitrogen and 0.4 for carbon [38].

Potential prey items to input into the mixing model were selected through a combination of the results of these stomach contents and published reports of North Atlantic diets (S1 Table). For species with particularly diverse diets, such as Atlantic cod and thorny skate, prey that represented over 5% of the weight and/or numbers were analyzed separately and all other reported prey items were combined into four functional groups (Table 1): pelagic invertebrates, benthic invertebrates, pelagic fish and demersal fish. To acknowledge that any predator is gape-limited, only prey that were less than 24% of the predator’s body mass were included in the model [91]. As not all individual weights were obtained, length-weight relations were used to determine approximate body masses for both predators and potential prey (S2 Table). For individual invertebrates which did not have size information, average species sizes were used.

The stable isotope mixing model to determine the percentage of the diets represented by key prey species was implemented using the MixSIAR package in R [92]. Three basic equations were utilized in the Bayesian isotope mixing model to determine the proportions of the diet occupied by each prey type [93]:

$$\left(\delta^{13}C_{1}-\delta^{13}C_{M}\right){\left[C\right]}_{1}f_{1,B}+\left(\delta^{13}C_{2}-\delta^{13}C_{M}\right){\left[C\right]}_{2}f_{y,B}+\cdots +\;\left(\delta^{13}C_{n}-\delta^{13}C_{M}\right){\left[C\right]}_{n}f_{n,B}=0$$ (5)

$$\left(\delta^{15}N_{1}-\delta^{15}N_{M}\right){\left[N\right]}_{1}f_{1,B}+\left(\delta^{15}N_{2}-\delta^{15}N_{M}\right){\left[N\right]}_{2}f_{2,B}+\cdots +\left(\delta^{15}N_{n}-\delta^{15}N_{M}\right){\left[N\right]}_{n}f_{n,B}=0$$ (6)

$$f_{1,B}+f_{2,B}+\cdots +f_{n,B}=1$$ (7)

Where *δ*^13^*C*_*n*_/*δ*^15^*N*_*n*_ represent the tissue isotopic values for a given prey item, *δ*^13^*C*_*M*_/*δ*^15^*N*_*M*_ the tissue isotopic values for the predator, [*C*]_*n*_ the carbon concentration of a given prey, [*N*]_*n*_ the nitrogen concentration of a given prey, and *f*_*n*,*B*_ the proportion of the predator’s diet represented by the given prey species. Size category (small, medium, or large) was included as a fixed variable for species-region combinations that demonstrated ontogenetic variation. Region- and species-specific IRIs calculated within this study were provided as a prior to these mixing models [94]. Some species-region combinations did not contain stomach data due to a high percentage of empty stomachs. In these few cases, an average IRI from other available regions was used. For cases where there are three or fewer potential prey sources, this model can provide exact contributions to the predator’s diet of each prey. For greater than three potential prey sources, fifty thousand repetitions of the mixing model were run to determine the approximate proportion of the diet each prey taxon occupies.

### Food web metrics

Several food web metrics were calculated to characterize the three constructed food webs. The total number of “nodes” or functional groups represents the network size. The “connectance” of the food web is the fraction of all possible predatory links that are realized and the ratio of trophic links within the food web over the square of the network size [92, 93]. For each species, several metrics were calculated to determine the importance of each prey. The “number of links per node” represents the number of predators feeding on that prey. The “average percent of predator diets” is the mean percentage of a given prey within the linked predator diets. The “relative link strength” is calculated as the sum of all the diet proportions contributed by a given prey taxon over the total summation of diet proportions for all food web links within a given region.

From the outcomes of the isotope mixing models and these food web metrics, we constructed simplified food webs for the predatory fish species. Within isotope biplot space, all the species were plotted with species showing ontogenetic variability in their isotopic signatures represented as a maximum of three points. Links between predatory species and their respective prey, identified through our stomach content analyses and reported predation in the literature, were plotted with the line width proportional to the importance to the predator’s diet. The proportion of the total linkage strength for each prey item as determined by summing all the linkage strengths from the prey item and dividing by the sum of all linkage strengths determined within the food web. Circles were drawn over the respective prey items in biplot space in the reconstructed upper foodweb with radiuses proportional to the magnitude of these total linkage strengths to indicate the relative importance of prey species as conduits of energy flow into the upper food web.

## Results

### Index of relative importance

Stomach content data from all fish species were collected to use as priors for the stable isotope mixing model. From the stomach contents alone, half of the percent IRIs of the three focus fish were made up of shrimp, snow crab, hyperiids, or capelin (Table 3, S3 Table). Shrimp consumption increased in the northern regions, representing an average of 19.2% IRI in the Bonavista Corridor and 33.4% in the Hawke Channel. Gammarids were also a common prey item of Hawke Channel fish, representing an average of 13.3% IRI. Pelagic invertebrates, particularly hyperiids, were found to be a dominant prey item in the Notre Dame Channel representing 58.6% IRI, particularly hyperiids and copepods representing an average 26.0% and 31.5% IRI. The Bonavista Corridor was noted to have the highest incidence of crab (8.5% IRI), polychaetes (16.7%), and pelagic fish (particularly capelin; 4.3%).

**Table 3:** Diet compositions of three highlighted predatory fish species presented as percent index of relative importance (IRI) within three regions: Hawke Channel (HC), Notre Dame Channel (NDC), or Bonavista Corridor (BC).

Species	Fractionation Coefficient (N/C)	Region	Prey species
**Atlantic Cod**	3.9 / -0.4	HC	Shrimp (72.2%), Demersal Fish (25.3%), Other Benthic Invertebrates (2.5%)
		NDC	Shrimp (98.4%), Snow Crab (0.4%), Hyperiid (0.3%), Demersal Fish (0.3%), Benthic Invertebrates (0.3%), Euphausiid (0.2%), Polychaete (0.1%)
		BC	Snow Crab (43.9%), Benthic Invertebrates (38.7%), Shrimp (11.9%), Polychaete (1.6%), Demersal Fish (1.6%), Checker Eelpout (1.0%), Hyperiid (0.8%), Pelagic Fish (0.5%)
**Greenland Halibut**	3.4 / 0.4	HC	Shrimp (73.1%), Gammarid (10.7%), Demersal Fish (8.0%), Benthic Invertebrates (3.9%), Copepod (1.7%), Capelin (0.4%), Pelagic Invertebrates (0.4%)
		NDC	Hyperiid (99.7%), Gammarid (0.1%), Shrimp (0.1%)
		BC	Capelin (82.9%), Shrimp (10.1%), Hyperiid (2.7%), Checker Eelpout (1.3%), Copepod (1.2%), Benthic Invertebrates (1.2%), Redfish (0.4%), Gammarid (0.3%)
**Redfish**	3.4 / 0.4	HC	Shrimp (76.3%), Copepod (14.7%), Hyperiid (5.5%), Euphausiid (2.0%), Capelin (1.0%), Benthic Invertebrates (0.4%)
		NDC	Shrimp (81.5%), Copepod (11.1%), Mysid (3.9%), Capelin (2.3%), Euphausiid (1.2%)
		BC	Shrimp (77.4%), Hyperiid (12.3%), Copepod (8.0%), Euphausiid (1.0%), Capelin (0.9%), Mysid (0.3%), Benthic Invertebrates (0.1%)

A fractionation coefficient was estimated from this stomach contents data to use in the isotope mixing model with the first number representing the nitrogen fractionation followed by the carbon fractionation.

### Simplified food web model

**Fig 2:**
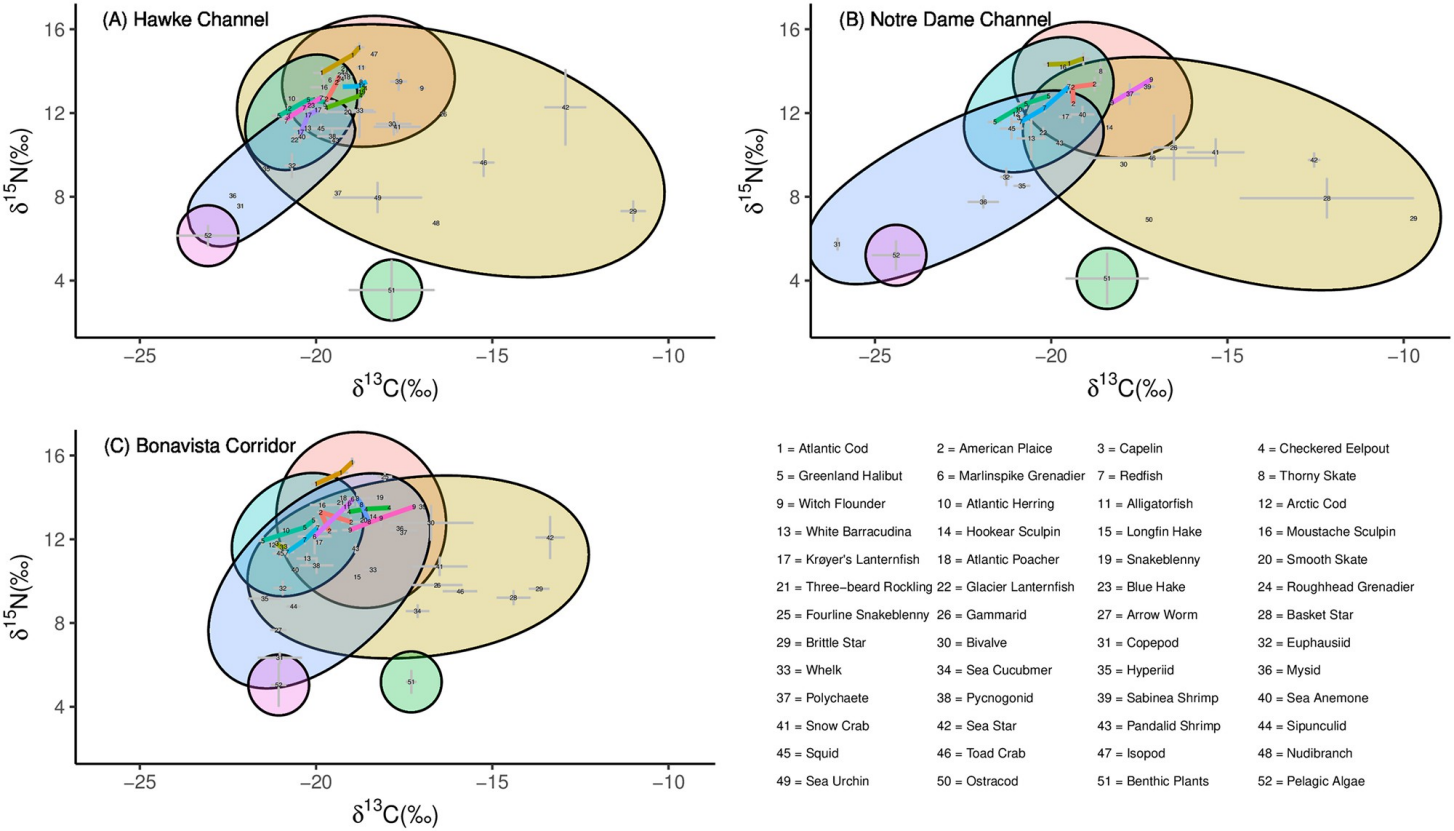
Stable carbon and nitrogen isotope values (mean ±SE) for dominant species within (A) the Hawke Channel, (B) the Notre Dame Channel, and (C) the Bonavista Corridor. Ovals represent the group categories of pelagic fish (green), benthic fish (red), pelagic invertebrates (blue), benthic invertebrates (yellow), pelagic algae (purple), and benthic plants (dark green). In cases where a species demonstrated ontogenetic variation in their isotopic signatures, these species are represented by three points connected by linear lines. Species abbreviations are defined in Table 1.

The mean and standard error of the *δ*^13^C and *δ*^15^N for each species relative to other members of the community are shown in Fig 2. Ellipses enclosing benthic invertebrates, demersal fish, pelagic invertebrates, and pelagic fish, representing bulk prey categories for species with particularly varied diets, were estimated using the Khachiyan algorithm for the computation of minimum volume enclosing ellipsoids [95]. The most nitrogen depleted values were observed in the pelagic algae and benthic plant material (Fig 2) and the highest trophic level species was the Atlantic cod. While the pelagic components of the ecosystem fell within a relatively small range of *δ*^13^C values, the benthic community was found to exhibit a wide spread of *δ*^13^C values (Fig 2). Within the benthic component of the food web invertebrates, particularly echinoderms, exhibited a larger range of carbon values than the fish species.

**Fig 3:**
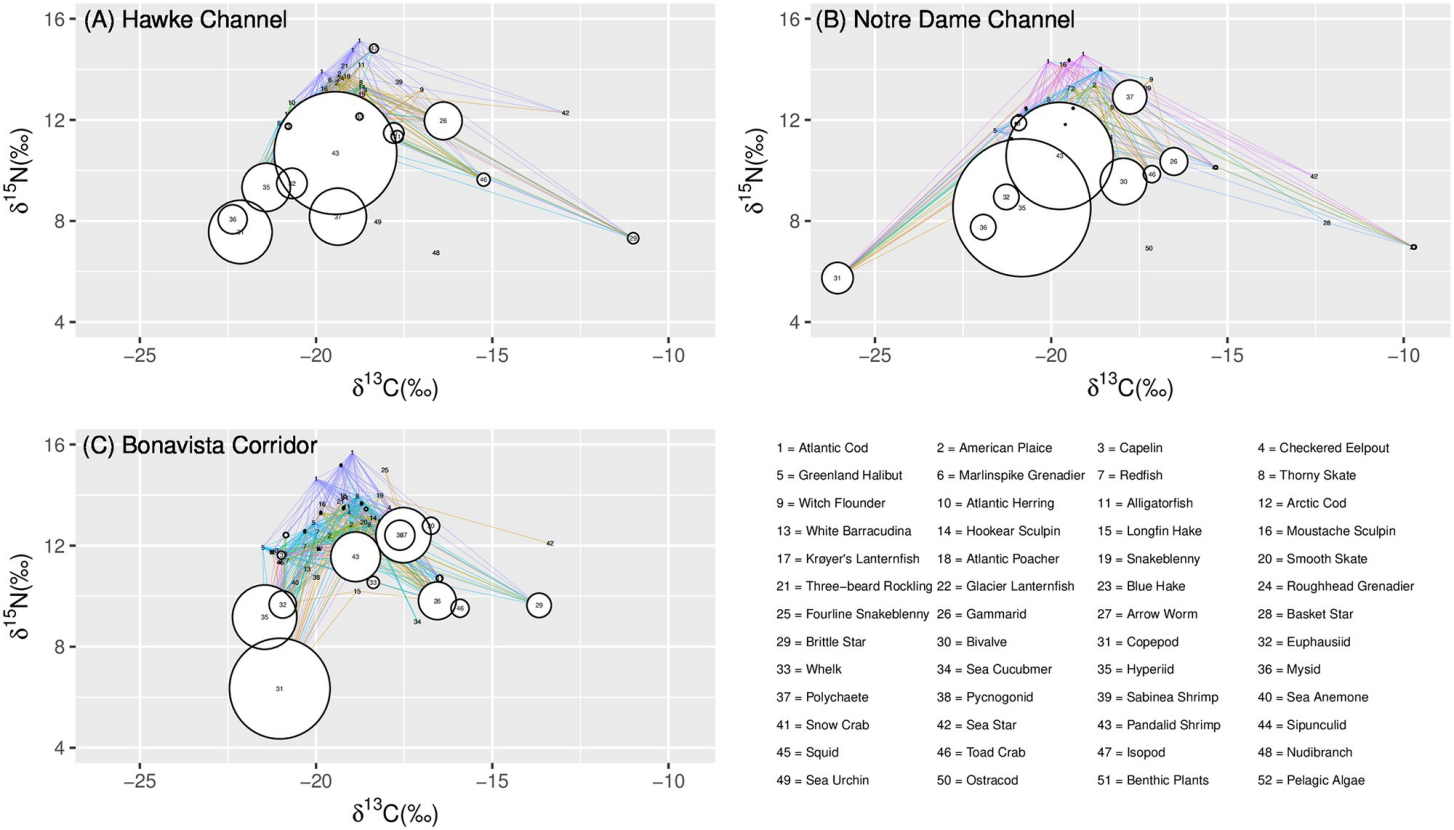
Simplified food webs with line widths indicating the linkage strength of the predatory interaction for (A) the Hawke Channel, (B) the Notre Dame Channel and (C) the Bonavista Corridor. Circles represent the relative linkage strength from the given prey species. Species from which a number of size classes were obtained will appear up to three times (for small, medium, and large size categories). Taxa abbreviations are defined in Table 1.

The food web for the Newfoundland and Labrador shelf regions, even when simplified, displayed numerous links (Fig 3). Each region consisted of between 31 and 43 observed trophic “nodes” with the Notre Dame Channel (Fig 3B) having the fewest nodes, likely lower due to less intensive sampling (Table 4). However, the highest latitude system had an ~10% increase in the number of links per prey species (Table 5), which is associated with increased connectance. Strong latitudinal increases were observed in the linkage strength of shrimp, more than doubling from 0.105 in the south to 0.264 in the north. Similarly, PPD increased from 21.2 to 38.1. In contrast, prey such as copepods (LS increasing from 0.135 to 0.216, PPD from 14.8 to 24), brittle stars (LS increasing from 0.021 to 0.050, PPD from 4.8 to 12) and fish species were more frequently consumed in the southern regions (Fig 3, Table 5). The Notre Dame Channel also saw increases in prey items from the center of the biplot space such as hyperiids (LS of 0.297, PPD of 37.3) and bivalves (LS of 0.098, PPD of 27.7) (Fig 3B).

**Table 4:** Three food web metrics calculated for the food webs in each region.

Region	Network Size	Links per Species	Connectance
**Hawke Channel**	42	4.40	0.105
**Notre Dame Channel**	31	3.94	0.127
**Bonavista Corridor**	43	3.93	0.091

**Table 5:** Food web link characteristics for the three focus regions.

Prey Species	Bonavista Corridor			Notre Dame Channel			Hawke Channel		
	#L	LS	PPD	#L	LS	PPD	#L	LS	PPD
**American Plaice**	3	0.001	1.4 (±0.2)	4	0.001	1.7 (±0.1)	0	0	0
**Arctic Cod**	9	0.002	3.2 (±1.5)	0	0	0	0	0	0
**Atlantic Cod**	8	0.001	1.0 (±2.0)	0	0	0	0	0	0
**Atlantic Herring**	14	0.008	1.2 (±1.3)	0	0	0	0	0	0
**Bivalve**	20	0.034	10.1 (±1.3)	13	0.098	27.7 (±3.5)	19	0.041	10.7 (±1.6)
**Brittle Star**	13	0.050	14.8 (±1.8)	12	0.006	1.4 (±0.8)	17	0.021	4.8 (±2.0)
**Capelin**	31	0.014	7.6 (±1.0)	14	0.030	6.2 (±1.6)	14	0.010	3.9 (±1.8)
**Checker Eelpout**	3	0.004	0.3 (±2.3)	0	0	0	0	0	0
**Copepod**	33	0.216	31.9 (±1.8)	24	0.065	6.9 (±1.2)	34	0.135	14.8 (±1.2)
**Euphausiid**	31	0.056	10.7 (±1.4)	23	0.052	7.8 (±2.1)	29	0.063	8.0 (±1.8)
**Gammarid**	34	0.079	11.7 (±1.8)	21	0.057	8.6 (±1.2)	31	0.079	10.9 (±1.7)
**Gastropod**	16	0.023	6.9 (±3.2)	0	0	0	12	0.012	3.7 (±4.9)
**Greenland Halibut**	6	0.001	2.1 (±0.2)	0	0	0	0	0	0
**Hyperiid**	32	0.137	25.1 (±2.1)	22	0.297	37.3 (±2.5)	24	0.102	17.4 (±1.9)
**Isopod**	0	0	0	0	0	0	11	0.016	7.4 (±4.5)
**Marlinspike**	6	0.001	2.0 (±0.2)	0	0	0	0	0	0
**Myctophiid**	13	0.001	2.5 (±2.6)	0	0	0	0	0	0
**Mysid**	30	0.062	13.2 (±1.1)	23	0.052	10.6 (±2.0)	35	0.060	6.1 (±1.2)
**Polychaete**	30	0.117	17.2 (±1.4)	18	0.071	11.4 (±2.0)	26	0.121	18.3 (±1.8)
**Shrimp**	30	0.105	21.2 (±1.4)	20	0.230	28.9 (±2.3)	28	0.264	38.1 (±2.5)
**Snow Crab**	13	0.011	10.1 (±1.2)	13	0.004	2.1 (±1.2)	14	0.023	7.5 (±3.4)
**Squid**	0	0	0	0	0	0.2 (±1.1)	10	0.009	3.7 (±1.2)
**Thorny Skate**	3	0.001	2.1 (±0.2)	0	0	0	0	0	0
**Toad Crab**	16	0.036	8.7 (±1.3)	11	0.034	6.9 (±4.8)	15	0.025	5.3 (±1.8)

#L (Number of Links) represents the number of predators preying on that species/group. LS represents relative link strength which is calculated as the sum of all the diet proportions contributed by a given prey taxon over the total summation of diet proportions for all food web links within a given region. For each prey species, the percent of each linked predator diet represented by that prey was averaged as PPD (average percent of predator diet) with standard error in parentheses.

### Trends among three focus species

While a variety of predatory fishes were analyzed as part of this study, we chose to focus on three abundant and economically important species. For details on additional abundant species, refer to S1–S6 Figs.

**Fig 4:**
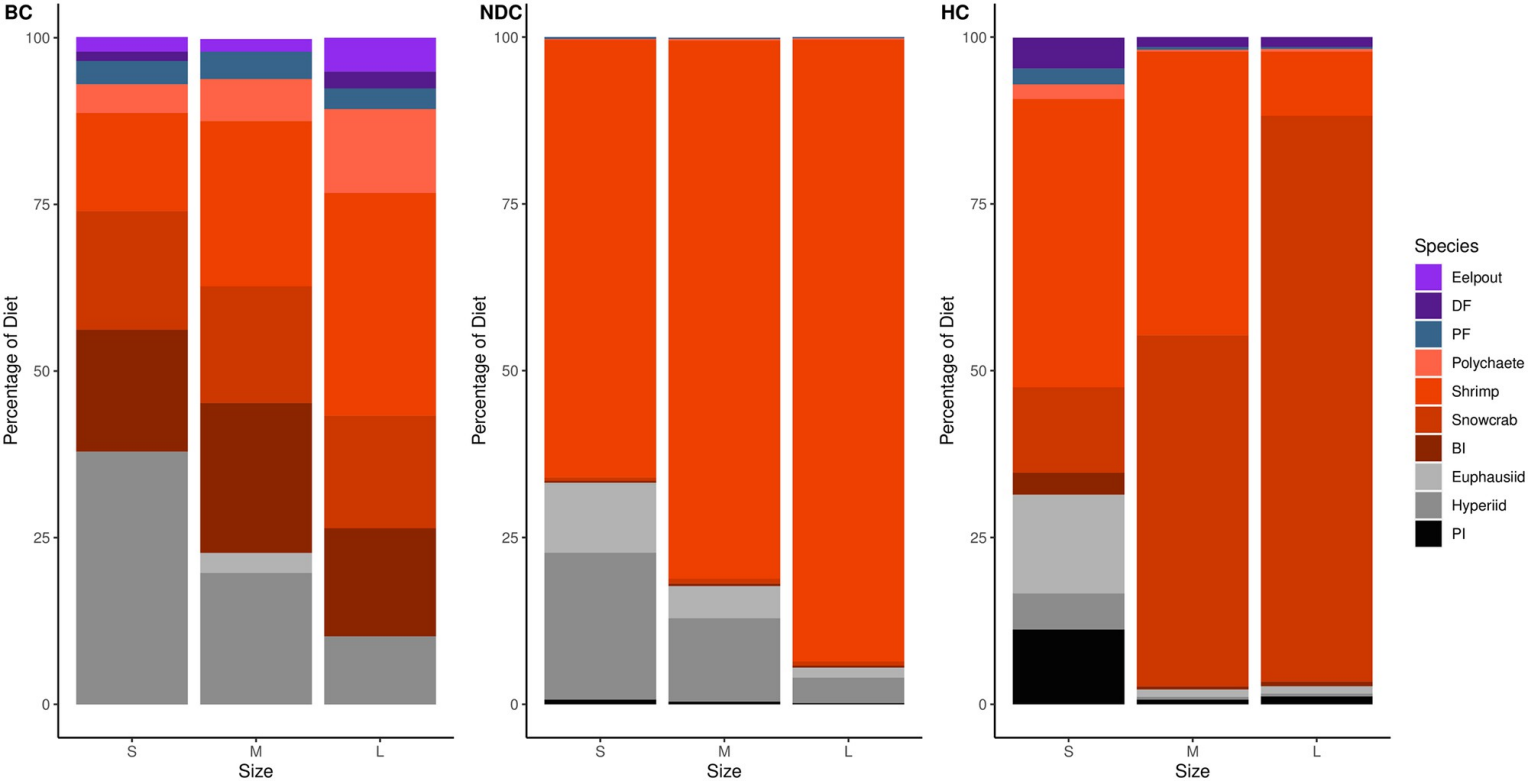
Atlantic cod diet composition percentages as determined from isotope mixing models. This figure is divided by predator size category (S for small, M for medium, L for large, see Table 2) and region (BC for Bonavista Corridor, NDC for Notre Dame Channel, HC for Hawke Channel). Prey taxa abbreviations are defined in Table 1.

Within Atlantic cod diets, in the BC, 75% consisted of shrimp, snow crab, polychaetes, hyperiids and benthic invertebrates; in the NDC: shrimp and hyperiids; in HC: shrimp, snow crab, euphausiids, and pelagic invertebrates (Fig 4). Across all three regions shrimp made up a quite substantial portion of cod diets increasing in the northern regions (Table 1). With increasing cod size, the contribution of zooplankton to the diet decreases and is replaced with benthic prey. Furthermore, snow crab, another economically important species in the region, was observed to make up quite a substantial portion of the diet in the Bonavista Corridor and dominate cod diets within the Hawke Channel (Fig 4). Demersal and pelagic fish make up a small portion of the diet in the Bonavista Corridor that was found to decrease in the other two regions (Fig 4).

**Fig 5:**
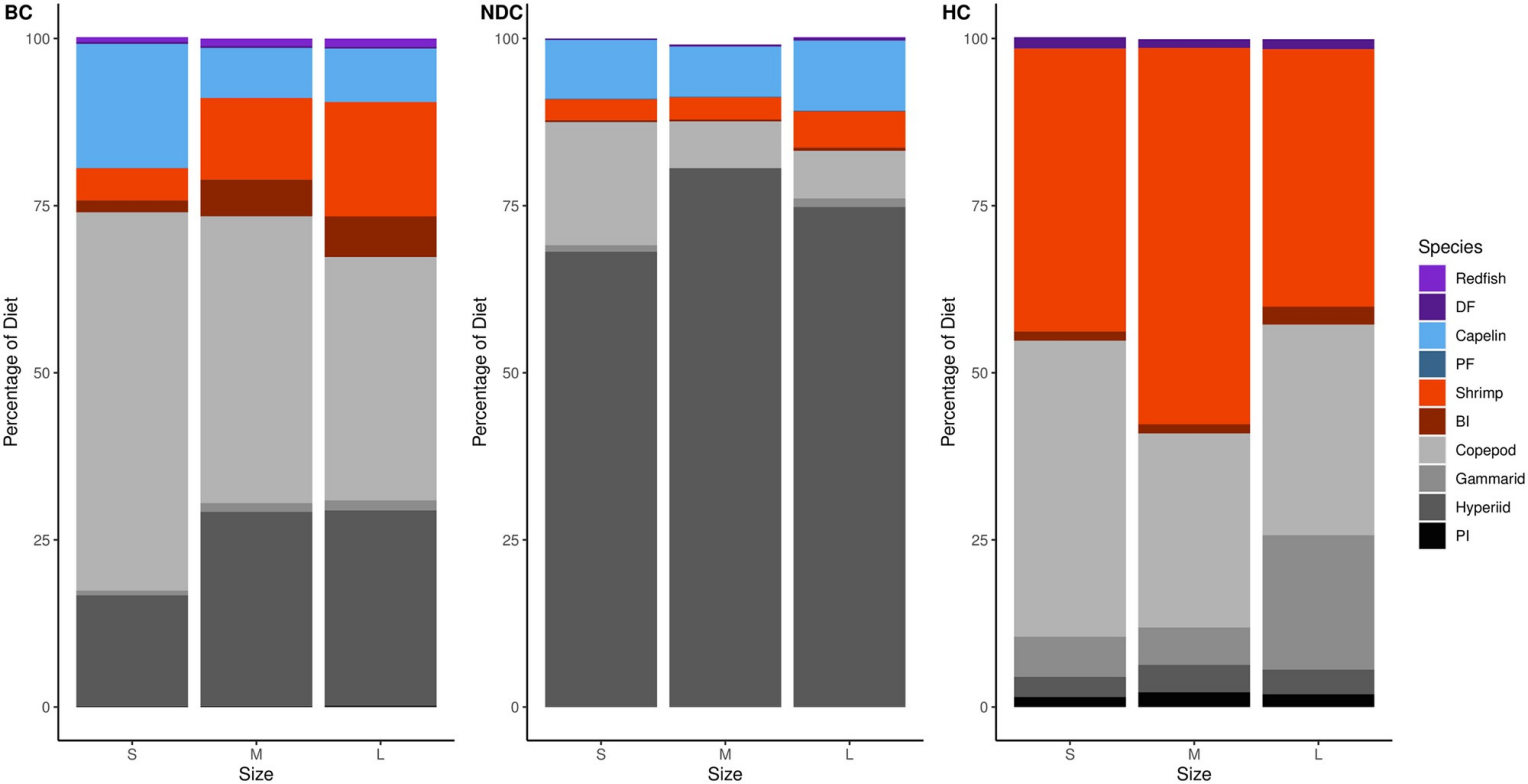
Greenland halibut diet composition percentages as determined from isotope mixing models. This figure is divided by predator size category (S for small, M for medium, L for large, see Table 2) and region (BC for Bonavista Corridor, NDC for Notre Dame Channel, HC for Hawke Channel). Prey taxa abbreviations are defined in Table 1.

In the Bonavista Corridor the diet of this fish was comprised mostly of copepods with generous contributions from hyperiids and capelin (Fig 5). The Notre Dame Channel saw a shift from a copepod-dominated diet to one comprised primarily of hyperiids. Finally, shrimp dominated the diets within Hawke Channel. With increasing Greenland halibut size, there was a decrease in zooplankton consumption (Fig 5).

**Fig 6:**
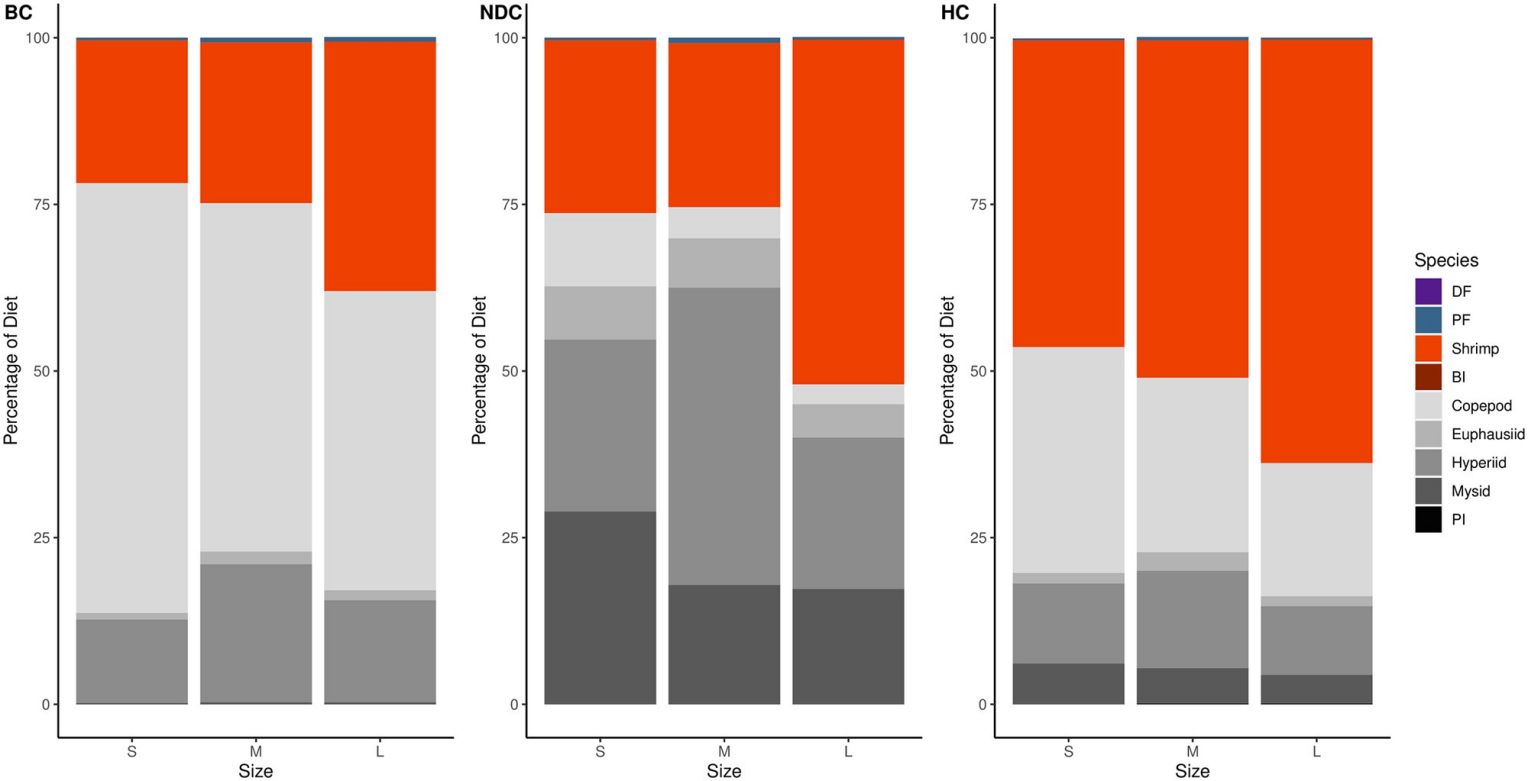
Redfish diet composition percentages as determined from isotope mixing models. This figure is divided by predator size category (S for small, M for medium, L for large, see Table 2) and region (BC for Bonavista Corridor, NDC for Notre Dame Channel, HC for Hawke Channel). Prey taxa abbreviations are defined in Table 1.

Redfish diets were relatively less complex in comparison to the previous two predators, consisting of mostly zooplankton and shrimp (Fig 6). The relative proportions of these two contributions varied among regions with higher contribution of shrimp with increasing latitude. Increases in shrimp consumption were also observed with ontogeny within all regions.

## Discussion

We report the results of within- and among-region variation in the Newfoundland and Labrador shelf marine ecosystem analyzed using isotope mixing models to examine whether regional variation in community overlap [47] and differential size-based community recovery rates [6] may be explained by differences in energy and nutrient flows through ecosystems recovering from overexploitation [5]. While every food web is inherently a simplification of reality, based on the results of both the stomach content and the stable isotope analyses, the importance of specific forage species changes markedly among the three analyzed regions. These findings have implications for management of both established invertebrate fisheries and recovering groundfish fisheries and further reveal the importance of spatial downscaling species interactions within large marine ecosystems.

Stable isotope ecology has proven to be an effective means of assessing energy flows as it directly attempts to track the path of rarer heavier atoms through the food web [39, 96, 97]. In addition to helping to identify and quantify key trophic interactions within the ecosystem, particularly robust to spatial variation in the importance of shrimp among regions, stable isotopes also provided insights into the underlying processes that are governing these ecosystems by resolving the relative importance of the pelagic and benthic components of the community. To demonstrate the importance of considering this regional variability in species interactions, we separated the food-webs by regions defined by Rose [66] and highlighted the regional variation in reconstructed diets observed in three economically important groundfish species. Although partitioning the entire study area into smaller sub-regions may initially seem unnecessary, if spatial similarity was observed, the three areas could simply be recombined [2]. However, we observed regional variation in both the stable isotope model outcomes and in the composition of the catches and the stomach contents. These findings and consistent, biogeographic differences in food web metrics along this latitudinal gradient parallels results from spatial partitioning of the Barents Sea ecosystem, where species interactions, food web metrics, and environmental conditions varied among four main food-web regions [2].

While observed extremes in the carbon values of invertebrates indicated either pelagic or benthic signatures, the fish species tended towards a central carbon isotope value. This pattern tends to became more pronounced with increasing fish size, suggesting a balance between the consumption of pelagic and benthic productivity. The balanced acquisition of pelagic and demersal productivity has recently been hypothesized to explain the dominance of large benthic demersal fishes over large pelagic fishes in boreal and temperate regions [98], while other comparative studies in this region have focused largely on the dynamics of single prey species [62, 99]. Whether this energy acquisition hypothesis also influences ecosystem recovery is an important question with potential implications for differential ecosystem recovery pathways. For example, recoveries of community size-structure are strongest in the Bonavista Corridor compared to the other two regions [6]. Given both this hypothesis and our reported patterns, further examinations of benthic-pelagic prey contributions to predator diets should be included in analyses of differential recovery within and among marine ecosystems.

To further explain benthic and pelagic differences in prey consumption among regions, each region was characterized by a few key prey species as revealed by the link strength between predators and prey in the food web models. The fish diets in the Bonavista Corridor are known to be the most diverse of these three regions from previous published research [11, 47]. This study further demonstrated that groundfish in this region show increased consumption of a variety of fish species (many of them pelagic forage fish), copepods, and brittle stars. Apart from the increased copepod and brittle star consumption, these observations result in overall higher trophic level of the top-level predators and facilitate strong contributions of both the pelagic and benthic portions of the food webs towards generalist feeders.

In contrast, the Notre Dame Channel was characterized by important prey species with mid-range carbon and nitrogen isotopic values: shrimp, bivalves and hyperiids. Relatively extreme carbon values were observed in this region among copepods and echinoderms. As periphyton develops, the *δ*^13^C has been observed to increase into the range which could explain the extreme carbon values observed in echinoderms that likely would at least partially predate on this periphyton [100–102]. Furthermore, spatial variation in these values could be explained with the composition and thickness of this periphyton. Enriched *δ*^13^C observed in echinoderms have also been explained as a result of metabolic processes and preferential selection of high *δ*^13^C prey such as diatoms [103–105]. Spatial variation in copepod carbon isotope ratios have been observed elsewhere yet have not been fully explained, though it has been proposed that they reflect long-term trends in primary productivity carbon combined with temporal metabolic variability and spatial variation in algal isotopic composition [106–108]. The stable isotope and the stomach content data, however, consistently demonstrated the importance of zooplankton and infauna to the diets of fish in this region.

Finally, the Hawke Channel food web was dominated by the consumption of shrimp, although polychaete consumption was also found to be relatively higher in this region even if it only comprised a small portion of the diet. The vast majority of species in this region were found to at least have shrimp in their stomach contents if not representing the majority of the contents, consistent with numerous other groundfish diet studies in this region [11, 109, 110]. These patterns and evident differences in food web metrics among regions reiterates the importance of spatial downscaling to quantify variation in species interactions within marine ecosystems [1, 2, 4, 5, 47].

Many of the known detritivore invertebrates have been noted to have relatively enriched carbon signatures. This finding is consistent with the results found in other studies of temperate and high-latitude systems [111, 112]. We were not able to obtain isotopic values for the detritus, but we may infer this food source would possess a more enriched nitrogen and carbon signature compared to the benthic plant material in order to account for the wide range of values in the benthic portion of the community [113–115].

Three abundant fish species of socioeconomic importance were analyzed in greater detail due to recent debates regarding their population status and interactions with other fisheries. The biomass of these predators is often greater in the southern region studies than the north [116–119] and represented the most abundant species caught in our bottom trawls [6, 47]. We observed that shrimp and/or crab often comprised a substantial portion of the diet with the proportion increasing with ontogeny (Figs 4–6).

Additionally, as northern regions show less diversity in diet and fish communities [47], shrimp and crab become increasingly important channels of nutrient flow through the food web. Shrimp and crab represent the two most economically dominant fisheries within the entire Newfoundland and Labrador region, with landed values of $196 million and $309 million in 2019, respectively (120These values greatly exceed the recent landed values of Atlantic cod ($21 million), redfish ($10 million), and Greenland halibut ($58 million) [120]. Thus, while shrimp and crab are presently the two most lucrative fisheries they also represent major prey for these groundfish species [e.g. 3, 121, 122], the impact of which depends on the respective species- and size-specific predator population sizes and regions.

Given these food-web links, demersal fish recovery would therefore likely negatively impact the present shellfish industries. Studies have shown that large aggregations of cod and shrimp don’t coexist in this ecosystem, with increases associated with decreases in the other [3, 4, 123–125]. This interaction is exacerbated by each species favoring differing environmental conditions such as ocean temperatures [4, 126–130]. Quantifying spatial variation in these interactions reverses the decades-old question of whether declines of Atlantic cod facilitated the expansion of shrimp fisheries [3, 10] to now reveal regions in which lucrative shrimp fisheries and associated industries and communities may be impacted most by demersal fish recovery [131].

Several food web metrics were presented in order to quantify these interactions. The network size was found to be similar in the Hawke Channel and the Bonavista Corridor, though lower in the Notre Dame Channel. We observed that between the Bonavista Corridor and the northern regions there was a distinct increase in the connectance. This increase in connectance is associated with decreased food chain lengths and high predator-prey mass ratios (PPMR) [132, 133]. The observation that fish consumption tends to be reduced in the northern regions while zooplankton and shrimp become a much more prominent part of the diet could explain these observations, and the recent observation that PPMR is much higher in the Hawke Channel than other regions [6]. The links per prey species were found to be greater in the northern regions. Given these regions show lower diversity, predators would be expected to be more likely to prey upon common species. This metric therefore confirms that food web complexity is reduced in the northern regions, driven primarily by observed decreases local species diversity in these regions [6, 11]. This food web metric, however, has been called into question as it has been noted to incorrectly characterize ecological trends when varying number of nodes are present between food webs [134]. Despite this caveat, we observe the largest difference between the two regions with comparable network size, indicating an underlying ecological process may be responsible for the observations. Numerous limitations exist when constructing isotope mixing models. Firstly, an assumption was made that all potential prey species are represented within the model. However, the survey gear types used inherently limited the species and the size ranges sampled in this study, and as such there are gaps that are not represented. An example of this is the absence of detritus in the analysis. As we did not have access to a grab sample, the detritus anticipated to make up a major component within the benthic food web needed to be left out. We could infer the approximate position within biplot space based on the positions of the detritivores but given the wide range of carbon and nitrogen isotope values among these detritivores we cannot conclusively state the exact range of detrital isotopic values. In regards to the energy input into the upper food web, however, the exclusion of detritus and periphyton is not anticipated to have a significant impact as it is unlikely that detritus makes up a significant portion of these predators’ diets. The absence of components such as detritus prevent us from constructing a complete food web, but should not inhibit our ability to analyze species interactions within the upper food web to understand key prey species within these communities.

Most taxa that were identified in the stomachs had representative isotopic values from the trawl data. While all the major taxa present within the stomachs (representing > 5% of the Atlantic cod stomach contents by weight), minor invertebrate taxa were sometimes not represented in the isotopic signature. The Bonavista Corridor was missing sea cucumbers (3.4% of cod stomach content biomass); Notre Dame Channel was missing gastropods, crabs (*Cancer* sp.), tunicates, bryozoans and sea urchins (all at less than 0.1% of cod and Greenland halibut stomach content biomass); and Hawke Channel was missing sipunculids (0.1% of American Plaice stomach content biomass) and bryozoans (0.01% of cod stomach content biomass). Given the limitations of trawl sampling of invertebrate communities, it is not unexpected to be missing taxa. The only fish species that was found in a stomach that was not observed in the trawl catches were grenadiers in the Notre Dame Channel (0.3% of stomach content biomass). With the exception of gastropods, all of the prior mentioned taxa would have been grouped in either “other benthic invertebrates” or “other demersal fish” categories. Since these underrepresented species occurred infrequently in limited quantities, it appears unlikely that their omission would impact the results of the isotope mixing model. We also chose not to sample these taxa from the stomachs themselves as digestion has been shown to influence isotopic compositions [135, 136].

The stomach contents were also used in combination with those from other published studies as a means of determining potential prey as inputs for the stable isotope mixing model. However, this method may introduce uncertainty regarding the detectability of different prey [137]. For example, prey types will have variable evacuation rates which could bias the results of stomach contents analysis [138] but combining this analysis with stable isotope analysis helps to resolve this bias. An alternative means of detection within the stomach would be to use stomach content DNA to determine prey diversity [139, 140]. However, such methods are also subject to errors in the detectability of prey due to DNA degradation and as such should be used in combination with other methods [141].

The isotope mixing models are also known to be highly sensitive to discrimination factors [142]. Yet the measurement of these factors can often be difficult for this kind of study. While extensive lab testing is often encouraged to determine these contributions [143], many factors will influence the exact value of these factors within a given tissue type, including temperature [86], feeding rate [86], isotopic values of the prey [84, 144], protein and fat content of the prey [88, 145] and approximate trophic position [146]. Considering this wide range of uncertainty, we initially attempted to estimate a discrimination factor based on our observed stomach content information and isotope values of the prey. However, this estimation sometimes produced unreasonable results likely due to the low sample sizes of stomachs for some species with a sometimes high percentage of empty stomachs. In these cases, we used the measure provided by Post [38] as an approximation with full knowledge that this estimate is likely a simplification of reality, yet robust enough to provide an interpretable outcome.

The number of potential prey sources were often quite high for many of the species analyzed. Previous work in this region identified over 100 different prey species within cod stomachs [11]. This number of potential prey sources is naturally beyond what the model can realistically handle. Group categories were created to account for minor contributions to the diets within stomach data in the present study and previously published studies. For species with particularly varied diets, this resulted in 14 prey categories as inputs into the model [97]. Although this situation is less than ideal, as over 7 prey categories are to be used with caution [92], such an approach would limit the analyses to our 4 group categories and three individual prey items for species with high diet variability. We therefore opted to increase the number of potential inputs at the tradeoff that the outputs are more likely to be confounded and the model less likely to converge. Even so, interpretable results and trends arose from the study with comparable results from repeated test runs.

Despite these uncertainties in the measures of the diet composition, the proportions represented by each prey item provide an indication of the relative importance of each prey item [97]. The isotope mixing models for more than 3 prey sources are based on probabilities of each prey item being selected and as such often have high variation as the resulting proportions are an average of 10,000 runs of the model. Therefore, instead of focusing on determining the exact proportions of each prey, we focused on determining key prey items for the food web as well as spatial variation in these trends. The use of stomach content information used as a prior helped to direct the measure of dietary proportions towards this end. However, these priors were not found to directly determine the outputs of the stable isotope mixing models but merely increased or decreased the likelihood of individual prey species being important to the predator. In several instances the priors presented to the model did not match the long-term isotopic signature of the predator. We interpreted these occurrences as the priors demonstrating short-term diet patterns that were not representative of the long-term diets of these fish. Alternatively, the short-term patterns may represent a switch from a winter to summer diet. This study was primarily focused on describing the winter and spring diets for the various members of the community. A complementary study for the summer and fall diets would help to determine if this mismatch of priors and isotope signatures represent a seasonal shift in diets.

The Newfoundland and Labrador marine ecosystems are still in a state of recovery following overexploitation beginning decades ago [3–6, 61, 62, 65, 82, 147]. Despite significant progress towards recovery, many groundfish populations have not yet reached reference points for commercial exploitation [5, 6, 119, 120], a process that can be rate limited by interspecific interactions [148]. Through consideration of species interactions, we have highlighted interactions between current and recovering fisheries to illustrate likely ecological and economic impacts of groundfish recoveries. If additional groundfish populations approach levels where exploitation may resume in a sustainable manner, ecosystem and trophic dynamic considerations such as those presented should be considered in management decisions in order to facilitate ecosystem productivity and recovery. Such information gaps as trophodynamics and how they vary spatially are essentials inputs to ecosystem-based models that interact with other gaps such as essential fish habitat and population dynamics to help build an ecosystem framework of management [16, 149–151].

In summary, along the Newfoundland and Labrador shelf, spatially variable recovery rates of groundfish stocks are associated with variation in trophic interactions. Through a combination of stable isotope and stomach content analyses, multispecies diets reconstructed the upper food web within three regions. While the southern-most region showed the greatest diversity in diets with multiple species being of near equal contribution as prey, the diets were dominated by only one or two species in the northern two regions. This finding demonstrates that diet diversity within the community is positively associated with recovery rates of groundfish species, if not directly influencing those rates. Recent concerns about the future of economically important Pandalid shrimp fisheries in the context of potential groundfish recoveries illustrates the need to quantify spatial variation in trophic interaction and key pathways of energy flow into the upper food web. Pandalid shrimp were a key food web node as a significant contribution to the diets of multiple fish species. Further comparison of the diets of three economically important fishes confirmed spatial variability in prey consumption, emphasizing the need to appreciate and incorporate fine-scale variation in trophic interactions and consideration of the roles of both target and non-target species when developing management advice.

## Supporting information

S1 Table List of prey species/groups included in the isotope mixing model for each predatory fish species. Determined from our stomach contents analyses and the presented studies from other researchers.
(DOCX)

S2 Table Length-weight relationships of representative fish species of Newfoundland and Labrador. (DOCX)

S3 Table Fractionation coefficients and prey groups analyzed for the isotope mixing models of the remaining species. Percent IRI is presented in parentheses.
(DOCX)

S4 Table Carbon and nitrogen stable isotope values and concentrations for all sampled organisms. Reported values represent means (± SD).
(DOCX)

S5 Table Isotope sample sizes by region and size category with food-containing stomach sample sizes. (DOCX)

S1 Fig American Plaice diet composition as determined from isotope mixing models divided by size category and region. (PDF)

S2 Fig Atlantic Herring diet composition as determined from isotope mixing models divided by size category and region. (PDF)

S3 Fig Capelin diet composition as determined from isotope mixing models divided by size category and region. (PDF)

S4 Fig Eelpout diet composition as determined from isotope mixing models divided by size category and region. (PDF)

S5 Fig Lanternfish diet composition as determined from isotope mixing models divided by size category and region. (PDF)

S6 Fig Thorny Skate diet composition as determined from isotope mixing models divided by size category and region. Functional group categories are designated by two letter abbreviations: PI for pelagic invertebrates, BI for benthic invertebrates, PF for pelagic fish, DF for demersal fish.
(PDF)
