# Supplementary material for: Spatial variation in food web structure in a recovering marine ecosystem
Source: PLoS One. 2022 May 20;17(5):e0268440. doi: 10.1371/journal.pone.0268440 (PMC9122200; doi:10.1371/journal.pone.0268440)
Supplement: S1 Table — Determined from our stomach contents analyses and the presented studies from other researchers. (DOCX) [file pone.0268440.s001.docx]

**S1 Table**

| Species | Scientific Name | Prey Species/Groups | Diet Studies |
| --- | --- | --- | --- |
| Alligatorfish | *Aspidophoroides monopterygius* | Copepods, Euphausiids, Gammarids, Hyperiids, Isopods, Mysids, Polychaetes | 152 - 153 |
| American Plaice | *Hippoglossoides platessoides* | Bivalve, Brittlestar, Capelin, Copepod, Gammarid, Gastropod, Hookear Sculpin, Hyperiid, Mysid, Polychaete, Redfish, Shrimp, Snow Crab, Toad Crab | 154 - 160 |
| Arctic Cod | *Boreogadus saida* | Americna Plaice, Brittlestars, Copepods, Euphausiids, Gammarids, Hyperiids, Mysids, Redfish, Shrimp | 161 - 166 |
| Atlantic Cod | *Gadus morhua* | Atlantic Herring, Capelin, Checkered Eelpout, Euphausiid, Hyperiid, Mysid, Polychaete, Redfish, Shrimp, Snow Crab, Benthic Invertebrates, Pelagic Invertebrates, Demersal Fish, Pelagic Fish. | 11, 51 - 53, 57, 105, 167 - 185 |
| Atlantic Herring | *Clupea harrengus harrengus* | Arrow Worms, Copepods, Euphausiids, Hyperiids, Mysids, Shrimp | 186 - 189 |
| Atlantic Hookear Sculpin | *Artediellus atlanticus* | Bivalves, Copepods,  Euphausiids,  Gammarids,  Mysids, Polychaetes | 83 |
| Atlantic Poacher | *Agonus decagonus* | Bivalves, Brittlestars, Copepods, Gammarids, Mysids, Polychaetes | 83 |
| Capelin | *Mallotus villosus* | Arrow Worms, Copepods, Euphausiid, Gammarid, Gastropod, Hyperiid, Mysid, Polychaete, Shrimp | 190 - 193 |
| Checkered Eelpout | *Lycodes vahlii* | Bivalves, Brittlestars, Copepods, Gammarids, Hyperiids, Polychaetes, Shrimp | 194 |
| Fourline Snakeblenny | *Eumesogrammus pracisus* | Euphausiids, Gammarids, Mysids, Polychaetes, Shrimp | 153 |
| Glacier Lanternfish | *Benthosema glaciale* | Arrow Worms, Copepods, Euphausiids, Gammarids, Hyperiids, Mysids, Shrimp | 83 |
| Greenland Halibut | *Reinhardtius hippoglossoides* | Atlantic Cod, Atlantic Herring, Capelin, Copepods, Checkered Eelpout, Gammarids, Hyperiid, Redfish, Shrimp, Squid, Benthic Invertebrates, Pelagic Invertebrates,  Demersal Fish,  Pelagic Fish | 58 - 61, 160, 195 - 197 |
| Krøyer’s Lanternfish | *Notoscopelus* sp. | Arrow Worm, Copepod, Euphausiids, Gammarids, Hyperiids, Mysid, Shrimp | 198 - 201 |
| Longfin Hake | *Urophycis chesteri* | Copepods, Euphausiids, Gammarids,  Hyperiids, Lanternfish, Shrimp | 202 - 204 |
| Marlinspike | *Nezumia bairdi* | Bivalves, Copepods, Euphausiids, Gammarids, Hyperiids, Mysids, Polychaetes, Shrimp, Toad Crab | 204 - 205 |
| Moustache Sculpin | *Triglops murrayi* | Copepods, Euphausiids, Gammarids, Mysids, Polychaetes, Shrimp, Toad Crab | 206 - 207 |
| Redfish | *Sebastes* sp. | Brittlestars, Capelin, Copepods, Euphausiids, Hyperiids, Lanternfishes, Mysids, Redfish, Shrimp, White Barracudina, Benthic Invertebrates, Pelagic Invertebrates, Demersal Fish, Pelagic Fish | 54 - 57, 156, 208 - 211 |
| Roughhead Grenadier | *Macrourus berglax* | Bivalves, Copepods, Euphausiids, Gammarids, Hyperiids, Mysids, Polychaetes, Shrimp, Toad Crab | 204 - 205 |
| Smooth Skate | *Raja senta* | Euphausiids, Gammarids, Mysids, Polychaetes, Redfish, Shrimp, Snow Crab, Toad Crab | 212 |
| Snakeblenny | *Lumpenus lumpretaeformis* | Bivalves, Copepods, Euphausiids, Gammarids, Mysids, Polychaetes, Sea Stars, Shrimp, Toad Crab | 213 |
| Thorny Skate | *Raja radiata* | Capelin, Copepods, Euphausiids, Gammarids, Polychaetes, Redfish, Shrimp, Snakeblennies, Snow Crab, Squid, Benthic Invertebrates, Pelagic Invertebrates, Demersal Fish, Pelagic Fish | 212, 214 - 216 |
| Threebeard Rockling | *Gaidropsarus ensis* | Arrow Worms, Bivalves, Euphausiids, Gammarids, Hyperiids, Mysids, Polychaetes, Shrimp | 217 - 219 |
| White Barracudina | *Notolepis rissoi* | Copepods, Capelin, Euphausiids, Hyperiids, Mysids, Shrimp | 153 |
| Witch Flounder | *Glyptocephalus cynoglossus* | Bivalves, Euphausiids, Gammarids, Gastropods, Polychaetes, Mysids | 160, 220 |

**Supplementary References**

152. Hutchings JA. Ecology and biodiversity of commercially unexploited marine fishes in the Northwest Atlantic. Final Report, Dalhousie University, Halifax, Nova Scotia; 2002.

153. Arbour JH, Avendaño P, Hutchings JA. Aspects of the ecology and life history of alligatorfish *Aspidophoroides monopterygius*. Environl Biol Fish 2010;87(4): 353-362.

154. Powles PM. Life history and ecology of American plaice (*Hippoglossoides platessoides* F.) in the Magdalen Shallows. J Fish Res Board Can 1965;22(2): 565-598.

155. Pitt TK. Food of American plaice (*Hippoglossoides platessoides*) from the Grand Bank, Newfoundland. J Fish Rese Board Can 1973;30(9): 1261-1273.

156. Canalejo FJP, Alvarez FJV, Arroyo AFA, Sanchez JMC. The feeding of American plaice (*Hippoglossoides platessodies*), redfish (*Sebastes marinus*) and cod (*Gadus morhua*) in the Flemish Cap during July 1988. NAFO SCR Doc 1989;89/45.

157. Keats DW. American plaice, *Hippoglossoides platessoides* (Fabricius), predation on green sea urchins, *Strongylocentrotus droebachiensis* (O.F. Muller), in eastern Newfoundland. J Fish Biol 1991;38: 67-72.

158. Zamarro J. Feeding behaviour of the American plaice (*Hippoglossoides platessoides*) on the southern Grand Bank of Newfoundland. Netherlands J Sea Res 1992;29(1-3): 229-238.

159. Martell DJ, McClelland G. Diets of sympatric flatfish, *Hippoglossoides platessoides*, *Pleuronectes ferrugineus*, *Pleuronectes americanus*, from Sable Island Bank, Canada. J Fish Biol 1994;44: 821-848.

160. Link JS, Bolles K, Milliken CG. The feeding ecology of flatfish in the Northwest Atlantic. J Northwest Atl Fish Sci 2002;30: 1-17.

161. Bohn A, McElroy RO. Trace Metals (As, Cd, Cu, Fe, and Zn) in Arctic cod, *Boreogadus saida*, and selected zooplankton from Strathcona Sound, Northern Baffin Island. J Fish Res Board Can 1976;33: 2836-2840.

162. Ajiad AM, Gjøsæter H. Diet of polar cod, *Boreogadus saida*, in the Barents Sea related to fish size and geographical distribution. ICES CM 1990; G:48.

163. Hobson KA, Welch HE. Determination of trophic relationships within a high arctic marine food web using Delta 13 C and Delta 15 N analysis. Mar Ecol Prog Ser 1992;84: 9-18.

164. Hop H, Welch HE, Crawford RE. Population structure and feeding ecology of arctic cod schools in the Canadian high Arctic. Am Fish S S 1997;19: 68-80.

165. Christiansen JS, Hop H, Nilssen EM, Joensen J. Trophic ecology of sympatric Arctic gadoids, *Arctogadus glacialis* (Peters, 1872) and *Boreogadus saida* (Lepechin, 1774), in NE Greenland. Polar Biol 2012;35(8): 1247-1257.

166. McNicholl DG, Walkusz W, Davoren GK, Majewski AR, Reist JD. Dietary characteristics of co-occurring polar cod (*Boreogadus saida*) and capelin (*Mallotus villosus*) in the Canadian Arctic, Darnley Bay. Polar Biol 2016;39(6): 1099-1108.

167. Popova OA. Some data on the feeding of cod in the Newfoundland area of the Northwest Atlantic. (Translated for U.S. Dept Int. and Nat. Sci. Found., Washington, D.C., by Israel Program for Scientific Translations, 1963) In: Soviet fisheries investigations in the Northwest Atlantic. VNIRO-PINRO Moscow. 1962: 228-248.

168. Templeman W. Some instances of cod and haddock behavior and concentrations in the Newfoundland and Labrador areas in relation to food. ICNAF Spec Publ. 1966;6: 449-461.

169. Turuk TN. Seasonal changes of cod feeding in the Labrador and Newfoundland areas in 1964-1966. Trudy PINRO. 1968;23 (Fisheries Research Board of Canada Translation Series No. 1937): 370-382.

170. Stanek E. Observations on food and feeding of cod (*Gadus morhua*) in Labrador, Newfondland and Nova Scotia waters. Pr Morsk Inst Rly 17, Ser A, 7-26.

171. Stanek E. The percentage of capelin in the stomach contents of cod in ICNAF Subareas 2 and 3. ICNAF Res Doc 1975/5, Ser No. 3433, 9 p.

172. Turuk TN. The daily food ration of the Labrador and Newfoundland cod. Trudy PINRO. 1976; 37(Fisheries and Marine Service Translation Series No. 4510): 19-25.

173. Turuk TN, Postolaky AI. Feeding and food relations of some fish species in the Labrador and Newfoundland areas. NAFO SCR Doc 1980;80/VI/70.

174. Lilly GR, Fleming AM. Size relationships in predation by Atlantic cod, *Gadus morhua*, on capelin, *Mallotus villosus*, and sand lance, *Ammodytes dubius*, in the Newfoundland area. NAFO Sci Coun Studies 1981;1: 41-45.

175. Lilly GR, Rice JC. Food of Atlantic cod (*Gadus morhua*) on the northern Grand Bank in spring. NAFO SCR Doc. 1983;83/IX/87.

176. Lilly GR. 1984. Annual variability in the diet of Atlantic cod (*Gadus morhua* L.) off southern Labrador and Northeast Newfoundland (Div. 2J + 3K) in autumn, 1977-82. NAFO SCR Doc 1984;84/79.

177. Lilly GR, Osborne DR. Predation by Atlantic cod (*Gadus morhua*) on short-finned squid (*Illex illevebrosus*) off eastern Newfoundland and in the northeastern Gulf of St. Lawrence. NAFO SCR Doc 1984;84/IX/108.

178. Lilly GR. Variability in the quantity of capelin and other prey in the stomachs of Atlantic cod off southern Labrador and Northeastern Newfoundland (NAFO Divisions 2J + 3K) during the autumns of 1978-85. NAFO SCR Doc 1986;86/80.

179. Methven DA, Piatt JF. Seasonal and annual variation in the diet of Atlantic cod (*Gadus morhua*) in relation to the abundance of capelin (*Mallotus villosus*) off eastern Newfoundland, Canada. ICES J Mar Sci 1989;45(2): 223-225.

180. Gerasimova OV, Albikovskaya LK, Kuzmin SA. A study of trophic interrelations between cod (*Gadus morhua*) and Capelin (*Mallotus villosus*) on the Newfoundland Shelf in spring and summer seasons of 1985-1991. NAFO Sci Coun Studies Doc 1992; 92/15.

181. Lilly GR. Predation by Atlantic cod on capelin on the southern Labrador and Northeast Newfoundland shelves during a period of changing spatial distributions. ICES Mar Sc 1994;198: 600-611.

182. Casas JM, Paz J. Recent changes in the feeding of cod (*Gadus morhua*) off the Flemish Cap, Newfoundland 1989-1993. ICES J Mar Sci 1996;53: 750-756.

183. Gerasimova OV, Kiseleva VM. Long-term variations in cod distribution and feeding on the Newfoundland shelf in spring and summer. NAFO Sci Coun Studies 1998;31: 79-110.

184. DeBlois EM, Rose GA. Cross-shoal variability in the feeding habits of migrating Atlantic cod (*Gadus morhua*). Oecologia 1996;108: 192-196.

185. Hanson JM, Chouinard GA. Diet of Atlantic cod in the southern Gulf of St. Lawrence as an index of ecosystem change, 1959-2000. J Fish Biol 2002;60: 902-922.

186. Legaré JEH, MacIellan DC. 1960. A qualitative and quantitative study of the plankton of the Quoddy region in 1957 and 1958 with special reference to the food of the herring. J Fish Res Board Can 17(3): 409-448

187. De Silva SS. Food and feeding habits of the herring *Clupea harengus* and the sprat *C. sprattus* in inshore waters of the west coast of Scotland. Mar Biol 1973;20: 282-290.

188. Dalpadado P, Ellertsen B, Melle W, Dommasnes A. Food and feeding conditions of Norwegian spring-spawning herring (*Clupea harengus*) through its feeding migrations. ICES J Mar Sci 2000;57: 843-857.

189. Langøy H, Nøttestad L, Skaret G, Broms C, Fernö A. Overlap in distribution and diet of Atlantic mackerel (*Scomber scombrus*), Norwegian spring-spawning herring (*Clupea harrengus*) and blue whiting (*Micronesistius poutassou*) in the Norwegian Sea during late summer. Mar Biol Res 2012;8: 442-460.

190. Vesin J-P, Leggett WC, Able KW. Feeding ecology of capelin (*Mallotus villosus*) in the estuary and western Gulf of St. Lawrence and its multispecies implications. Can J Fish Aquat Sci 1981;38(3): 257-267.

191. O'Driscoll RL, Parsons MJD, Rose GA. Feeding of capelin (*Mallotus villosus)* in Newfoundland waters. Sarsia 2001;86(3): 165-176.

192. Hedeholm R, Grønkjær P, Rysgaard S. Feeding ecology of capelin (*Mallotus Villosus* Müller) in West Greenland waters. Polar Biol 2012;35: 1533-1543.

193. Dalpadado P, Mowbray F. Comparative analysis of feeding ecology of capelin from two shelf ecosystems, off Newfoundland and in the Barents Sea. Prog Oceanogr 2013;114: 97-105.

194. Albert OA. Distribution, population structure and diet of silvery pout (*Gadiculus argenteus thori* J. Schmidt), poor cod (*Trisopterus minutus minutus* (L.)), four-bearded rockling (*Rhinonemus cimbrius* (L.)), and Vahl’s eelpout (*Lycodes vahlii gracilis* Reinhardt) in the Norwegian Deep. Sarsia 1993;78(2): 141-154.

195. Rodríguez-Marín E, Punzón A. Feeding patterns of Greenland halibut (*Reinhardtius hippoglossoides)* in Flemish Pass (Northwest Atlantic). NAFO Sci Coun Studies 1995;23: 43-54.

196. Orr DC, Bowering WR. A multivariate analysis of food and feeding trends among Greenland halibut (*Reinhardtius hippoglossoides*) sampled in Davis Strait, during 1986. ICES J Mar Sci 1997;54(5): 819-829.

197. Hovde SC, Albert OT, Nilssen EM. Spatial, seasonal and ontogenetic variation in diet of Northeast Arctic Greenland halibut (*Reinhardtius hippoglossoides*). ICES J Mar Sci 2002;59: 421-437.

198. Gjøsæter J. Life history and ecology of the myctophid fish *Notoscopelus elongates kroeyeri* from the northeast Atlantic. FiskDir Skr Ser HavUnders 1981;17: 133-152.

199. Podrazhanskaya SG. Feeding habits of mesopelagic species of fish and estimation of plankton graze in the northwest Atlantic. NAFO Sci Coun Studies 1993;19: 79-85.

200. García-Seoane E, Dalpadado P, Vázquez A. Feeding ecology of the glacier lanternfish *Benthosema glaciale* (Actinopterygii, Myctophidae) in the Flemish Cap (North Atlantic Ocean). Hydrobiologia 2013;717: 133-146.

201. Bernal A, Olivar MP, Maynou F, Fernández de Puelles ML. Diet and feeding strategies of mesopelagic fishes in the western Mediterranean. Prog Oceanograph 2015;135: 1-17

202. Wenner CA. Biology of the longfin hake, *Phycis chesteri*, in the western north Atlantic. Biol Oceanogr. 1983;3(1): 41-75.

203. Methven DA, McKelvie DS. Distribution of *Phycis chesteri* (Pisces: Gadidae) on the Grand Bank and Labrador Shelf. Copeia 1986;4: 886-891.

204. Pérez-Rodríguez A, Koen-Alonso M, González-Iglesias C, Saborido-Rey F. Analysis of common trends in the feeding habits of main demersal fish species on the Flemish Cap. NAFO SCR Doc 2011;11/77.

205. Savvatimsky PI. Distribution and biology of common grenadier (*Nezumia bairdi*) from trawl surveys in the Northwest Atlantic, 1969-83. NAFO Sci Coun Studies. 1989;13: 53-58.

206. Musick JA, Able KW. Occurrence and spawning of the sculpin *Triglops murrayi* (Pisces, Cottidae) in the Gulf of Maine. J Fish Res Board Can 1969;26: 473-475.

207. Atkinson EG, Percy JA. Diet comparison among demersal marine fish from the Canadian arctic. Polar Biol 1992;11: 567-573.

208. Steele DH. The redfish (*Sebastes marinus* L.) in the western Gulf of St. Lawrence. J Fish Res Board Can. 1957;14(6): 899-924.

209. Yanulov KP. Feeding habits of “beaked” redfish (*Sebastes mentalla* Travin) in the Newfoundland-Labrador area. Int Comm Northwest Atl Fish Redb. 1962; 3: 132-140.

210. Albikovskaya LK, Gerasimova OV. Food and feeding patterns of cod (*Gadus morhua* L.) and beaked redfish (*Sebastes mentella* Travin) on Flemish Cap. NAFO Sci Coun Studies 1993;19: 31-39.

211. Savenkoff C, Morin B, Chabot D, Castonguay M. Main prey and predators of redfish (*Sebastes* spp.) in the northern Gulf of St. Lawrence during the mid-1980s, mid-1990s, and early 2000s. Can Tech Rep Fish Aquat Sci. 2006; 2648.

212. McEachran JD, Boesch DF, Musick JA. Food division within two sympatric species-pairs of skates (Pisces: Rajidae). Mar Biol 1976;35: 301-317.

213. Demontigny F, Ouellet P, Sirois P, Plourde S. Zooplankton prey selection among three dominant icthyoplankton species in the northwest Gulf of St. Lawrence. J Plankton Res 2012;34(3): 221-235.

214. Templeman W. Stomach contents of the Thorny Skate, *Raja radiata*, from the Northwest Atlantic. J Northw Atl Fish Sci. 1982;3: 123-126.

215. Garrison LP. Spatial and dietary overlap in the Georges Bank groundfish community. Can J Fish Aquat Sci 2000;57: 1679-1691.

216. Skjæraasen JE, Bergstad OA. Distribution and feeding ecology of *Raja radiata* in the northeastern North Sea and Skagerrak (Norwegian Deep). ICES J Mar Sci. 2000;57: 1249-1260.

217. Houston KA, Haedrich RL. Food habits and intestinal parasites of deep demersal fishes from the upper continental slope east of Newfoundland, northwest Atlantic Ocean. Mar Biol 1986;92: 563-574.

218. Keats DW, Steele DH. The fourbeard rockling, Enchelyopus cimbrius (L.), in eastern Newfoundland. J Fish Biol 1990;37: 803-811.

219. Lampart-Kałużniacka M, Heese T. Studies on the biology of non-commercial species, based on the example of the fourbeard rockling Enchelyopus cimbrius (L., 1766) (Gadiformes: Lotidae) in the southern Baltic. Annales Universitatis Mariae Curie-Skłodowska Lublin-Polonia 2015;70(1): 43-60.

220. Scott JS. Digenetic trematode parasites and food of witch flounder (*Glyptocephalus cynoglossus* (Walbaum, 1792)) from the Scotian Shelf and Gulf of St. Lawrence. Fish Mar Serv Res Dev Tech Rep. 1976;618.
