# Supplementary material for: Spatial variation in food web structure in a recovering marine ecosystem
Source: PLoS One. 2022 May 20;17(5):e0268440. doi: 10.1371/journal.pone.0268440 (PMC9122200; doi:10.1371/journal.pone.0268440)
Supplement: S2 Table — (DOCX) [file pone.0268440.s002.docx]

**S2 Table**

| Species | Length-Weight Relation | Reference |
| --- | --- | --- |
| Alligatorfish | $W=0.0029*L^{3}$ | 221 |
| American Plaice | $W=0.0036*L^{3.305}$ | 222 |
| Arctic Cod | $W=0.0119*L^{2.76}$ | 223 |
| Atlantic Cod | $W=0.0081*L^{3.044}$ | 224 |
| Atlantic Herring | $W=0.0097*L^{2.96}$ | 225 |
| Atlantic Hookear Sculpin | $W=0.02*L^{2.85}$ | 226 |
| Atlantic Poacher | $W=0.0043*L^{2.98}$ | 221 |
| Capelin | $W=0.0042*L^{3.11}$ | 221 |
| Checker Eelpout | $W=0.0017*L^{3.27}$ | 221 |
| Fourline Snakeblenny | $W=0.0042*L^{3.24}$ | 221 |
| Glacier Lanternfish | $W=0.0054*L^{3.08}$ | 221 |
| Greenland Halibut | $W=0.005*L^{3.1804}$ | 227 |
| Krøyer Lanternfish | $W=0.0054*L^{3.08}$ | 221 |
| Longfin Hake | $W=0.0104*L^{2.8226}$ | 222 |
| Marlin-spike | $W=0.0254*L^{2.89}$ | 221 |
| Moustache Sculpin | $W=0.0032*L^{3.46}$ | 221 |
| Redfish | $W=0.0247*L^{2.9364}$ | 222 |
| Roughhead Grenadier | $W=0.1851*L^{2.7542}$ | 222 |
| Smooth Skate | $W=0.02*L^{2.85}$ | 222 |
| Snakeblenny | $W=0.0164*L^{2.09}$ | 221 |
| Thorny Skate | $W=0.0436*L^{2.8611}$ | 222 |
| Three-beard Rockling | $W=0.007*L^{2.977}$ | 221 |
| White Barracudina | $W=0.0003*L^{3.58}$ | 221 |
| Witch Flounder | $W=0.0008*L^{3.497}$ | 228 |

**Supplementary References**

221. Alpoim R, de Melo AA, Bañon R, Casas M, Cerviño S, Martín I, Murau H, et al. 2002. Distribution and main characteristic of fish species on Flemish Cap based on the 1988-2002 EU-Surveys in July. NAFO SCR Doc 2002;02/72.

222. Paz X, Román E. Length/weight relationships for some species of fish encountered in the Northwest Atlantic (NAFO regulatory area: Divisions 3L, 3M, and 3NO). NAFO SCR Doc 1997; 97/15.

223. Crawford RE, Jorgenson JK. Quantitative studies of arctic cod (*Boreogadus saida*) schools: Important energy stores in the Arctic food web. Arctic 1996;49(2): 181-193.

224. Árnason T, Björnsson B, Steinarsson A. Allometric growth and condition factor of Atlantic cod (*Gadus morhua*) fed to satiation: effects of temperature and body weight. J Appl Icthyol 2009;25(4): 401-406.

225. Wigley SE, McBride HM, McHugh NJ. Length-weight relationships for 72 fish species collected during NEFSC research vessel bottom travel surveys, 1992-99. NOAA Tech Memorandum. 2003;NMFS-NE-171.

226. Greenstreet SPR, Rossberg AG, Fox CJ, Le Quesne WJF, Blasdale T, Boulcott P, et al. Demersal fish biodiversity: species-level indicators and trends-based targets for the Marine Strategy Framework Directive. ICES J Mar Sci 2012;69: 1789-1801.

227. Román E, Paz X. 1997. Length/weight relationships for Greenland halibut, *Reinhardtius hippoglossoides*, from northwest Atlantic (NAFO regulatory area: Divisions 3L, 3M and 3NO). NAFO SCR Doc. 1997;97/16.

228. Bowering WR, Stansbury DE. Regressions of weight on length for witch flounder, *Glyptocephalus cynoglossus*, of the Eastern Newfoundland Area. J Northw Atl Fish Sci 1984;5: 105-106.
