# Supplementary material for: Spatial variation in food web structure in a recovering marine ecosystem
Source: PLoS One. 2022 May 20;17(5):e0268440. doi: 10.1371/journal.pone.0268440 (PMC9122200; doi:10.1371/journal.pone.0268440)
Supplement: S3 Table — Percent IRI is presented in parentheses. (DOCX) [file pone.0268440.s003.docx]

**S3** **Table**

| Species | Fractionation Coefficient | Region | Prey species analyzed |
| --- | --- | --- | --- |
| Alligatorfish | 4.20 (± 0.30) / 0.10 (± 0.03) | HC | Gammarid (87.9%), Euphausiid (12.1%) |
|  |  | BC | Copepod (72.4%), Polychaete (26.3%) |
| American Plaice | 3.40 (± 0.98) / 0.40 (± 1.30) | HC | Polychaete (65.8%), Brittlestar (17.8%), Toad Crab (6.3%), Hookear Sculpin (5.2%), Gammarid (2.3%), Shrimp (1.2%), Mysid (0.8%), Bivalve (0.3%), Copepod (0.3%) |
|  |  | NDC | Toad Crab (67.8%), Polychaete (24.8%), Gammarid (7.4%) |
|  |  | BC | Shrimp (44.9%), Bivalve (29.7%), Polychaete (18.3%), Gastropod (3.5%), Redfish (2.2%), Snow Crab (0.7%), Gammarid (0.6%) |
| Arctic Cod | 3.43 (± 0.34) / 0.54 (± 0.05) | HC | Copepod (43.8%), Shrimp (40.4%), Gammarid (15.8%) |
|  |  | NDC | Copepod (85.0%), Hyperiid (12.9%), Shrimp (1.6%), Euphausiid (0.4%), Gammarid (0.1%) |
|  |  | BC | Hyperiid (61.0%), Copepod (38.3%), Shrimp (0.4%), Gammarid (0.2%), Mysid (0.1%) |
| Atlantic Cod | 3.40 (± 0.18) / -0.40 (± 0.15) | HC | Shrimp (72.2%), Demersal Fish (25.3%), Other Benthic Invertebrates (2.5%) |
|  |  | NDC | Shrimp (98.4%), Snow Crab (0.4%), Hyperiid (0.3%), Demersal Fish (0.3%), Benthic Invertebrates (0.3%), Euphausiid (0.2%), Polychaete (0.1%) |
|  |  | BC | Snow Crab (43.9%), Benthic Invertebrates (38.7%), Shrimp (11.9%), Polychaete (1.6%), Demersal Fish (1.6%), Checker Eelpout (1.0%), Hyperiid (0.8%), Pelagic Fish (0.5%) |
| Atlantic Herring | 3.35 (± 0.17) / 0.26 (0.01) | HC | No food-containing stomachs. |
|  |  | NDC | Hyperiid (94.2%), Euphausiid (4.1%), Copepod (1.5%), Shrimp (0.2%) |
|  |  | BC | Hyperiid (100%) |
| Atlantic Poacher | 3.90 (± 0.30) / 0.40 (± 0.03) | HC | Gammarid (73.4%), Shrimp (15.1%), Polychaete (11.6%) |
|  |  | BC | Gammarid (79.3%), Copepod (15.1%), Mysid (5.2%), Euphausiid (0.4%) |
| Blue Hake | 3.40 (± 0.98) / 0.40 (± 1.30) | HC | No prey-containing stomachs |
| Capelin | 3.40 (± 0.98) / 0.40 (± 1.30) | HC | Copepod (75.0%), Mysid (14.8%), Hyperiid (9.5%), Euphausiid (0.4%), Polychaete (0.2%) |
|  |  | NDC | Copepod (80.8%), Hyperiid (18.6%), Mysid (0.2%), Shrimp (0.2%), Euphausiid (0.1%), Gammarid (0.1%), Gastropod (0.1%) |
|  |  | BC | Copepod (88.4%), Hyperiid (9.6%), Euphausiid (1.3%), Mysid (0.5%), Shrimp (0.2%) |
| Checkered Eelpout | 3.40 (± 0.98) / 0.40 (± 1.30) | HC | Polychaete (69.1%), Brittlestar (24.1%), Copepod (3.3%), Shrimp (2.4%), Bivalve (0.6%), Gammarid (0.6%) |
|  |  | BC | Polychaete (79.7%), Copepod (7.1%), Hyperiid (7.0%), Gammarid (6.1%) |
| Fourline Snakeblenny | 2.90 (± 0.17) / -0.20 (± 0.10) | BC | Polychaete (100%) |
| Glacier Lanternfish | 2.57 (± 0.13) / 0.92 (± 0.07) | HC | Copepod (74.0%), Gammarid (13.0%), Shrimp (13.0%) |
|  |  | NDC | Copepod (98.7%), Mysid (0.8%), Hyperiid (0.3%), Shrimp (0.3%) |
| Greenland Halibut | 3.40 (± 0.98) / 0.40 (± 1.30) | HC | Shrimp (73.1%), Gammarid (10.7%), Demersal Fish (8.0%), Benthic Invertebrates (3.9%), Copepod (1.7%), Capelin (0.4%), Pelagic Invertebrates (0.4%) |
|  |  | NDC | Hyperiid (99.7%), Gammarid (0.1%), Shrimp (0.1%) |
|  |  | BC | Capelin (82.9%), Shrimp (10.1%), Hyperiid (2.7%), Checker Eelpout (1.3%), Copepod (1.2%), Benthic Invertebrates (1.2%), Redfish (0.4%), Gammarid (0.3%) |
| Atlantic Hookear Sculpin | 3.00 (± 0.30) / 0.90 (± 0.10) | HC | Bivalve (72.4%), Copepod (27.7%) |
|  |  | NDC | Polychaete (66.7%), Copepod (16.7%), Gammarid (16.7%) |
|  |  | BC | Copepod (63.0%), Gammarid (19.5%), Polychaete (9.5%), Mysid (4.9%), Euphausiid (3.1%) |
| Krøyer’s Lanternfish | 2.74 (± 0.14) / 0.92 (± 0.05) | HC | Copepod (55.2%), Shrimp (23.1%), Hyperiid (18.7%), Euphausiid (1.7%), Mysid (1.3%), Gammarid (0.1%) |
|  |  | NDC | Copepod (49.3%), Shrimp (36.2%), Hyperiid (13.8%), Mysid (0.8%) |
|  |  | BC | Copepod (69.2%), Hyperiid (25.2%), Mysid (3.3%), Euphausiid (2.3%), Arrow Worm (0.1%) |
| Longfin Hake | 3.40 (± 0.98) / 0.40 (± 1.30) | BC | No prey-containing stomachs |
| Marlinspike | 3.10 (± 0.30) / 0.00 (± 0.01) | HC | Toad Crab (42.9%), Copepod (30.4%), Shrimp (17.0%), Hyperiid (9.8%) |
|  |  | BC | Mysid (48.8%), Bivalve (29.0%), Polychaete (17.3%), Gammarid (3.5%), Shrimp (1.4%) |
| Moustache Sculpin | 2.80 (± 0.30) / 0.40 (± 0.08) | HC | Shrimp (65.5%), Polychaete (12.3%), Toad Crab (9.6%), Copepod (6.3%), Gammarid (6.3%) |
|  |  | NDC | Hyperiid (70.0%), Gammarid (15.4%), Toad crab (8.4%), Polychaete (6.1%) |
|  |  | BC | Shrimp (32.5%), Mysid (22.5%), Copepod (22.5%), Gammarid (22.5%) |
| Redfish | 3.40 (± 0.98) / 0.40 (± 1.30) | HC | Shrimp (76.3%), Copepod (14.7%), Hyperiid (5.5%), Euphausiid (2.0%), Capelin (1.0%), Benthic Invertebrates (0.4%) |
|  |  | NDC | Shrimp (81.5%), Copepod (11.1%), Mysid (3.9%), Capelin (2.3%), Euphausiid (1.2%) |
|  |  | BC | Shrimp (77.4%), Hyperiid (12.3%), Copepod (8.0%), Euphausiid (1.0%), Capelin (0.9%), Mysid (0.3%), Benthic Invertebrates (0.1%) |
| Roughhead Grenadier | 3.00 (± 0.30) / 0.40 (± 0.01) | HC | Copepod (46.2%), Gammarid (23.7%), Polychaete (21.4%), Shrimp (4.9%), Brittle Star (1.3%), Snow Crab (0.9%), Euphausiid (0.8%), Mysid (0.6%) |
| Smooth Skate | 1.40 (± 0.25)/ 0.50 (± 0.02) | HC | Shrimp (49.5%), Euphausiid (43.1%), Polychaete (4.0%), Gammarid (3.3%) |
|  |  | BC | Shrimp (72.1%), Toad Crab (27.9%) |
| Snakeblenny | 4.1 (± 0.17) / 0.4 (± 0.10) | HC | No prey-containing stomachs |
|  |  | BC | Toad Crab (100%) |
| Thorny Skate | 2.46 (± 0.25) / 0.22 (± 0.02) | HC | Shrimp (75.2%), Polychaete (10.1%), Snow Crab (9.0%), Euphausiid (1.3%), Gammarid (1.3%), Benthic Invertebrates (1.3%), Copepod (0.9%), Squid (0.5%), Capelin (0.1%), Demersal Fish (0.1%), Pelagic Invertebrates (0.1%) |
|  |  | NDC | No prey-containing stomachs |
|  |  | BC | Shrimp (29.0%), Euphausiid (27.2%), Polychaete (11.2%), Capelin (7.0%), Snow Crab (7.0%), Pelagic Invertebrate (6.3%), Gammarid (5.3%), Benthic Invertebrates (2.7%), Snakeblenny (1.7%), Demersal Fish (1.4%), Copepod (1.0%), Redfish (0.2%), Squid (0.1%) |
| Threebeard Rockling | 3.4 (± 0.34) / 0.5 (± 0.04) | HC | No prey-containing stomachs |
|  |  | BC | Shrimp (72.9%), Hyperiid (8.4%), Polychaete (7.3%), Mysid (4.5%), Euphausiid (3.4%), Gammarid (3.4%) |
| White Barracudina | 3.40 (± 0.98) / 0.40 (± 1.30) | HC | Shrimp (71.8%), Copepod (19.8%), Euphausiid (4.9%), Capelin (1.9%), Hyperiid (1.2%), Gammarid (0.2%), Mysid (0.2%) |
|  |  | NDC | Shrimp (61.8%), Copepod (34.3%), Hyperiid (1.8%), Mysid (1.6%), Euphausiid (0.4%) |
|  |  | BC | Copepod (80.1%), Shrimp (18.6%), Euphausiid (1.1%), Hyperiid (0.3%) |
| Witch Flounder | 3.4 (± 0.60) / -0.1 (± 0.01) | HC | No prey-containing stomachs |
|  |  | NDC |  |
|  |  | BC | Polychaete (79.3%), Gammarid (13.2%), Euphausiid (5.2%), Bivalve (1.2%), Mysid (1.1%) |
