# Supplementary material for: Spatial variation in food web structure in a recovering marine ecosystem
Source: PLoS One. 2022 May 20;17(5):e0268440. doi: 10.1371/journal.pone.0268440 (PMC9122200; doi:10.1371/journal.pone.0268440)
Supplement: S4 Table — Reported values represent means (± SD). (DOCX) [file pone.0268440.s004.docx]

**S4 Table**

| Species Number | Common Name | Region | Size | $\boldsymbol{\delta}^{\boldsymbol{13}}$C (lipid corrected) | $\boldsymbol{\delta}^{\boldsymbol{15}}\boldsymbol{N}$ | [C] | [N] |
| --- | --- | --- | --- | --- | --- | --- | --- |
| 1 | Atlantic Cod | HC | L | -18.92  (± 0.16) | 15.14  (± 0.25) | 46.26  (± 0.43) | 14.42  (± 0.14) |
|  |  |  | M | -19.09  (± 0.08) | 14.78  (± 0.07) | 47.18  (± 0.56) | 14.60  (± 0.19) |
|  |  |  | S | -19.86  (± 0.21) | 14.04  (± 0.27) | 47.19  (± 0.24) | 14.37  (± 0.13) |
|  |  | NDC | L | -19.09  (± 0.17) | 15.58  (± 0.31) | 47.43  (± 0.24) | 14.57  (± 0.08) |
|  |  |  | M | -19.49  (± 0.15) | 14.81  (± 0.16) | 46.80  (± 0.37) | 14.46  (± 0.10) |
|  |  |  | S | -20.10  (± 0.13) | 14.14  (± 0.10) | 45.90  (± 0.75) | 14.22  (± 0.26) |
|  |  | BC | L | -18.98  (± 0.08) | 15.68  (± 0.22) | 47.45  (± 0.42) | 14.69  (± 0.14) |
|  |  |  | M | -19.29  (± 0.10) | 15.18  (± 0.28) | 47.44  (± 0.19) | 14.64  (± 0.08) |
|  |  |  | S | -20.01  (± 0.12) | 14.64  (± 0.11) | 47.03  (± 0.16) | 14.24  (± 0.09) |
| 2 | American Plaice | HC | L | -19.35  (± 0.12) | 13.78  (± 0.14) | 48.20  (± 0.64) | 15.06  (± 0.20) |
|  |  |  | M | -19.42  (± 0.09) | 13.45  (± 0.17) | 46.82  (± 0.42) | 14.59  (± 0.11) |
|  |  |  | S | -19.71  (± 0.26) | 12.57  (± 0.30) | 48.22  (± 0.69) | 14.12  (± 0.43) |
|  |  | NDC | L | -18.77  (± 0.16) | 13.38  (± 0.35) | 47.90  (± 0.28) | 14.76  (± 0.04) |
|  |  |  | M | -19.39  (± 0.12) | 13.24  (± 0.14) | 46.81  (± 0.28) | 14.54  (± 0.09) |
|  |  |  | S | -19.38  (± 0.06) | 12.46  (± 0.21) | 47.52  (± 0.47) | 14.65  (± 0.15) |
|  |  | BC | L | -19.01  (± 0.28) | 12.83  (± 0.24) | 47.79  (± 0.38) | 14.54  (± 0.14) |
|  |  |  | M | -19.87  (± 0.10) | 13.30  (± 0.22) | 48.24  (± 0.48) | 14.87  (± 0.13) |
|  |  |  | S | -19.63  (± 0.23) | 12.40  (± 0.22) | 47.22  (± 0.34) | 14.30  (± 0.14) |
| 3 | Capelin | HC |  | -20.79  (± 0.09) | 11.75  (± 0.15) | 49.15  (± 1.32) | 13.09  (± 0.48) |
|  |  | NDC |  | -20.92  (± 0.14) | 11.88  (± 0.16) | 48.39  (± 0.30) | 13.32  (± 0.35) |
|  |  | BC | L | -20.15  (± 0.10) | 11.66  (± 0.13) | 49.17  (± 1.06) | 13.24  (± 0.52) |
|  |  |  | M | -20.99  (± 0.04) | 11.62  (± 0.04) | 48.57  (± 1.44) | 13.74  (± 0.16) |
|  |  |  | S | -21.55  (± 0.23) | 11.80  (± 0.36) | 45.04  (± 2.54) | 12.97  (± 0.68) |
| 4 | Checkered Eelpout | HC | L | -18.65  (± 0.05) | 13.28  (± 0.05) | 47.46  (± 0.71) | 14.45  (± 0.62) |
|  |  |  | M | -18.73  (± 0.12) | 12.85  (± 13.23) | 47.18  (± 0.75) | 14.24  (± 0.26) |
|  |  |  | S | -19.71  (± 0.16) | 12.25  (± 0.28) | 47.99  (± 0.81) | 14.17  (± 0.35) |
|  |  | BC | L | -17.92  (± 0.08) | 13.51  (± 0.09) | 47.83  (± 0.49) | 14.68  (± 0.11) |
|  |  |  | M | -18.58  (± 0.17) | 13.45  (± 0.22) | 48.33  (± 0.33) | 14.82  (± 0.18) |
|  |  |  | S | -19.06  (± 0.16) | 13.31  (± 0.13) | 45.50  (± 1.27) | 13.86  (± 0.45) |
| 5 | Greenland Halibut / Turbot | HC | L | -19.75  (± 0.15) | 12.52  (± 0.11) | 54.78  (± 1.42) | 10.81  (± 0.75) |
|  |  |  | M | -20.23  (± 0.07) | 12.64  (± 0.11) | 52.41  (± 1.12) | 12.33  (± 0.45) |
|  |  |  | S | -21.06  (± 0.20) | 11.87  (± 0.08) | 47.63  (± 0.61) | 14.00  (± 0.15) |
|  |  | NDC | L | -20.07  (± 0) | 12.82  (± 0) | 47.21  (± 0) | 14.08  (± 0) |
|  |  |  | M | -20.72  (± 0.24) | 12.45  (± 0.25) | 51.89  (± 1.60) | 12.63  (± 0.61) |
|  |  |  | S | -21.60  (± 0.20) | 11.58  (± 0.13) | 50.42  (± 1.25) | 12.37  (± 0.43) |
|  |  | BC | L | -20.07  (± 0.11) | 12.91  (± 0.17) | 48.60  (± 2.69) | 11.18  (± 0.74) |
|  |  |  | M | -20.32  (± 0.14) | 12.57  (± 0.17) | 51.48  (± 0.54) | 12.50  (± 0.24) |
|  |  |  | S | -21.52  (± 0.12) | 11.93  (± 0.21) | 49.40  (± 1.42) | 13.91  (± 0.45) |
| 6 | Marlinspike Grenadier | HC |  | -19.60  (± 0.06) | 13.59  (± 0.10) | 47.83  (± 0.41) | 14.75  (± 0.10) |
|  |  | BC | L | -18.96  (± 0.17) | 13.93  (± 0.13) | 48.42  (± 0.37) | 14.91  (± 0.08) |
|  |  |  | M | -19.05  (± 0.05) | 13.73  (± 0.10) | 47.88  (± 0.59) | 14.57  (± 0.22) |
|  |  |  | S | -20.04  (± 0.48) | 12.14  (± 0.85) | 48.20  (± 0.52) | 14.08  (± 0.58) |
| 7 | Redfish | HC | L | -19.86  (± 0.17) | 12.72  (± 0.21) | 48.18  (± 0.39) | 14.33  (± 0.15) |
|  |  |  | M | -20.35  (± 0.13) | 12.25  (± 0.21) | 48.78  (± 0.39) | 14.05  (± 0.29) |
|  |  |  | S | -20.85  (± 0.20) | 11.70  (± 0.21) | 48.85  (± 0.83) | 14.22  (± 0.53) |
|  |  | NDC | L | -19.51  (± 0.14) | 13.25  (± 0.32) | 47.25  (± 0.58) | 14.49  (± 0.19) |
|  |  |  | M | -20.19  (± 0.07) | 12.25  (± 0.21) | 48.43  (± 0.46) | 14.54  (± 0.10) |
|  |  |  | S | -20.86  (± 0.23) | 11.58  (± 0.25) | 48.09  (± 0.322) | 13.86  (± 0.26) |
|  |  | BC | L | -19.96  (± 0.09) | 12.55  (± 0.23) | 48.08  (± 0.43) | 13.94  (± 0.15) |
|  |  |  | M | -20.32  (± 0.10) | 11.97  (± 0.21) | 48.09  (± 0.50) | 14.04  (± 0.11) |
|  |  |  | S | -20.82  (± 0.19) | 11.39  (± 0.25) | 47.35  (± 0.70) | 13.80  (± 0.16) |
| 8 | Thorny Skate | HC |  | -18.73  (± 0.08) | 13.38  (± 0.14) | 44.14  (± 0.32) | 15.52  (± 0.25) |
|  |  | NDC |  | -18.60  (± 0.15) | 14.00  (± 0.46) | 44.72  (± 0.77) | 16.63  (± 0.19) |
|  |  | BC | L | -18.83  (± 0.56) | 13.95  (± 0.50) | 45.86  (± 2.08) | 15.33  (± 0.94) |
|  |  |  | M | -18.71  (± 0.09) | 13.67  (± 0.15) | 44.09  (± 0.33) | 16.33  (± 0.18) |
|  |  |  | S | -18.50  (± 0.10) | 12.83  (± 0.23) | 44.15  (± 1.08) | 15.90  (± 0.50) |
| 9 | Witch Flounder | HC |  | -16.89  (± 0.12) | 13.19  (± 0.02) | 48.27  (± 0.17) | 14.87  (± 0.03) |
|  |  | NDC | L | -17.17  (± 0.05) | 13.60  (± 0.02) | 46.90  (± 0.11) | 14.50  (± 0.07) |
|  |  |  | M | -18.27  (± 0) | 12.50  (± 0) | 48.77  (± 0) | 14.72  (± 0) |
|  |  | BC | L | -17.22  (± 0.06) | 13.56  (± 0.12) | 43.73  (± 2.45) | 13.60  (± 0.73) |
|  |  |  | M | -18.16  (± 0.24) | 13.02  (± 0.45) | 47.58  (± 0.16) | 14.12  (± 0.29) |
|  |  |  | S | -19.03  (± 0.23) | 12.45  (± 0.15) | 46.17  (± 1.15) | 13.76  (± 0.41) |
| 10 | Atlantic Herring | HC |  | -20.75  (± 0.07) | 12.54  (± 0.10) | 52.72  (± 0.49) | 11.75  (± 0.62) |
|  |  | NDC |  | -20.96  (± 0.11) | 12.10  (± 0.16) | 49.76  (± 0.86) | 13.76  (± 0.34) |
|  |  | BC |  | -20.86  (± 0.14) | 12.42  (± 0.08) | 51.08  (± 1.03) | 13.27  (± 0.34) |
| 11 | Alligatorfish | HC |  | -18.73  (± 0.13) | 14.18  (± 0.13) | 44.36  (± 2.08) | 11.92  (± 0.48) |
|  |  | BC |  | -18.36  (± 0.78) | 13.09  (± 0.59) | 41.12  (± 3.51) | 11.44  (± 1.24) |
| 12 | Arctic Cod | HC |  | -20.81  (± 0.07) | 12.23  (± 0.15) | 47.46  (± 0.25) | 14.20  (± 0.13) |
|  |  | NDC |  | -21.26  (± 0.05) | 11.73  (± 0.09) | 47.21  (± 0.26) | 14.15  (± 0.08 |
|  |  | BC |  | -21.00  (± 0.04) | 11.86  (± 0.06) | 46.52  (± 0.31) | 13.89  (± 0.10) |
| 13 | White Barracudina | HC |  | -20.24  (± 0.10) | 11.28  (± 0.16) | 55.72  (± 2.88) | 11.22  (± 0.78) |
|  |  | NDC |  | -20.57  (± 0.41) | 10.79  (± 1.02) | 51.02  (± 5.72 | 10.81  (± 0.67) |
|  |  | BC |  | -20.25  (± 0.30) | 11.08  (± 0.28) | 56.45  (± 2.07) | 9.69  (± 0.73) |
| 14 | Atlantic Hookear Sculpin | HC |  | -18.71  (± 0.09) | 13.31  (± 0.40) | 45.98  (± 0.81) | 13.84  (± 0.38) |
|  |  | NDC |  | -18.36  (± 0) | 11.32  (± 0) | 39.02  (± 0) | 10.60  (± 0) |
|  |  | BC |  | -18.38  (± 0.05) | 13.09  (± 0.14) | 44.22  (± 1.62) | 13.40  (± 0.54) |
| 15 | Longfin Hake | BC |  | -18.84  (NA) | 10.20  (NA) | 42.68  (NA) | 10.62  (NA) |
| 16 | Moustache Sculpin | HC |  | -19.78  (± 0.44) | 13.23  (± 0.42) | 48.11  (± 0.47) | 14.38  (± 0.24) |
|  |  | NDC |  | -19.67  (± 0.06) | 14.20  (± 0.10) | 49.58  (± 1.09) | 14.39  (± 0.27) |
|  |  | BC |  | -19.83  (± 0.35) | 12.14  (± 0.24) | 47.25  (± 1.02) | 13.82  (± 0.08) |
| 17 | Krøyer’s Lanternfish | HC | L | -19.94  (± 0.21) | 12.15  (± 0.10) | 51.42  (± 1.66) | 12.84  (± 0.36) |
|  |  |  | M | -20.23  (± 0.07) | 11.90  (± 0.25) | 49.84  (± 0.46) | 12.98  (± 0.65) |
|  |  |  | S | -20.45  (± 0.01) | 10.98  (± 0.10) | 52.60  (± 2.04) | 12.09  (± 0.59) |
|  |  | NDC |  | -19.60  (± 0.18) | 11.82  (± 0.10) | 58.11  (± 0.77) | 9.56  (± 0.47) |
|  |  | BC |  | -19.93  (± 0.12) | 11.86  (± 0.09) | 55.06  (± 0.77) | 11.32  (± 0.50) |
| 18 | Atlantic Poacher | HC |  | -19.13  (± 0.15) | 13.74  (± 0.25) | 52.00  (± 2.73) | 12.55  (± 1.13) |
|  |  | BC |  | -19.24  (± 0.08) | 13.97  (± 0.17) | 47.31  (± 1.54) | 12.01  (± 0.48) |
| 19 | Snakeblenny | HC |  | -18.71  (± 0) | 13.12  (± 0) | 51.11  (± 0) | 15.06  (± 0) |
|  |  | BC |  | -18.19  (± 0.21) | 13.99  (± 0.20) | 50.13  (± 0.74) | 14.38  (± 0.03) |
| 20 | Smooth Skate | HC |  | -19.10  (0.09) | 12.05  (0.20) | 44.31  (± 0.39) | 15.94  (± 0.23) |
|  |  | BC |  | -18.65  (± 0) | 12.92  (± 0) | 44.76  (± 0) | 16.69  (±0) |
| 21 | Three-beard Rockling | HC |  | -19.18  (± 0) | 13.89  (±0) | 47.18  (± 0) | 14.13  (± 0) |
|  |  | BC |  | -19.29  (± 0.24) | 13.69  (± 0.29) | 46.91  (± 0.59) | 14.41  (± 0.24) |
| 22 | Glacier Lanternfish | HC |  | -20.60  (± 0.05) | 10.82  (± 0.21) | 55.20  (± 1.83) | 11.03  (± 0.55) |
|  |  | NDC |  | -20.23  (± 0.05) | 11.07  (± 0.25) | 57.29  (± 0.65) | 10.45  (± 0.29) |
| 23 | Blue Hake | HC |  | -20.14 (±0) | 12.38  (±0) | 50.22  (± 0) | 14.40  (± 0) |
| 24 | Roughhead Grenadier | HC |  | -19.31  (± 0.17) | 13.69  (± 0.26) | 41.54  (± 5.13) | 12.75  (± 1.58) |
| 25 | Fourline Snakeblenny | BC |  | -18.05  (± 0.21) | 15.00  (± 0.20) | 47.77  (± 0.22) | 14.77  (± 0.26) |
| 26 | Gammarid Amphipod | HC |  | -16.39  (±0) | 11.96  (±0) | 35.10  (± 0) | 8.09  (± 0) |
|  |  | NDC |  | -16.52  (± 0) | 10.35  (± 0) | 32.89  (± 0) | 8.25  (± 0) |
|  |  | BC |  | -16.56  (± 0.71) | 9.82  (± 2.09) | 32.15  (± 3.03) | 6.93  (± 1.79) |
| 27 | Arrow Worm | BC |  | -21.08  (± 0.23) | 7.67  (± 0.05) | 39.56  (± 9.73) | 8.63  (± 0.30) |
| 28 | Basket Star | NDC |  | -12.18  (± 0) | 7.93  (± 0) | 20.96  (± 0) | 2.97  (± 0) |
|  |  | BC |  | -14.40  (± 0.48) | 9.21  (± 0.36) | 20.21  (± 0.68) | 2.88  (± 0.08) |
| 29 | Brittle Star | HC |  | -11.00  (± 0.36) | 7.32  (± 0.51) | 18.32  (± 0.61) | 2.71  (± 0.09) |
|  |  | NDC |  | -9.71  (± 0) | 6.97  (± 0) | 17.61  (± 0) | 2.16  (± 0) |
|  |  | BC |  | -13.67  (± 0.29) | 9.64  (± 0.10) | 24.60  (± 9.77) | 5.05  (± 3.56) |
| 30 | Bivalve | HC |  | -17.80  (± 0.50) | 11.48  (± 0.55) | 31.43  (± 1.30) | 7.65  (± 0.39) |
|  |  | NDC |  | -17.95  (± 0) | 9.56  (± 0) | 32.00  (± 0) | 8.80  (± 0) |
|  |  | BC |  | -16.74  (± 1.20) | 17.78  (± 0.86) | 40.62  (± 7.82) | 10.71  (± 3.90) |
| 31 | Copepod | HC |  | -22.15  (± 0) | 4.17  (± 0) | 38.41  (± 0) | 7.56  (± 0) |
|  |  | NDC |  | -26.06  (± 0.04) | 5.74  (± 0.30) | 47.78  (± 3.07) | 3.14  (± 0.15) |
|  |  | BC |  | -21.03  (± 0.63) | 6.34  (± 0.28) | 45.06  (± 2.02) | 8.40  (± 0.62) |
| 32 | Euphausiid | HC |  | -20.69  (± 0.47) | 9.49  (± 0.21) | 50.02  (± 3.37) | 9.58  (± 0.60) |
|  |  | NDC |  | -21.27  (± 0.17) | 8.95  (± 0.43) | 46.17  (± 0.63) | 10.52  (± 0.17) |
|  |  | BC |  | -20.95  (± 0.26) | 9.67  (± 0.35) | 46.90  (± 1.46) | 10.59  (± 0.45) |
| 33 | Whelk | HC |  | -18.77  (± 0.39) | 12.13  (± 1.32) | 44.01  (± 0.26) | 11.77  (± 0.56) |
|  |  | BC |  | -18.38  (± 0.27) | 10.54  (± 0.15) | 46.19  (± 1.10) | 11.16  (± 0.29) |
| 34 | Sea Cucumber | BC |  | -17.12  (± 0.34) | 8.57  (± 0.32) | 23.00  (± 2.18) | 5.03  (± 1.35) |
| 35 | Hyperiid Amphipod | HC |  | -21.42  (± 0) | 9.33  (± 0) | 39.04  (± 0) | 8.49  (± 0) |
|  |  | NDC |  | -20.83  (± 0.23) | 8.52  (± 0.10) | 41.70  (± 2.11) | 7.79  (± 0.34) |
|  |  | BC |  | -21.46  (± 0.47) | 9.17  (± 0.18) | 38.58  (± 4.24) | 7.98  (± 0.40) |
| 36 | Mysid | HC |  | -22.37  (± 0) | 8.07  (± 0) | 37.57  (± 0) | 4.62  (± 0) |
|  |  | NDC |  | -21.93  (± 0) | 7.76  (± 0) | 38.70  (± 0) | 8.98  (± 0) |
|  |  | BC |  | -17.62  (± 0.10) | 12.41  (± 0.10) | 39.26  (± 1.80) | 9.44  (± 0.60) |
| 37 | Polychaete | HC |  | -19.38  (± 0) | 8.17  (± 0) | 43.82  (± 0) | 9.79  (± 0) |
|  |  | NDC |  | -17.77  (± 0.29) | 12.90  (± 0.51) | 39.41  (± 2.89) | 11.32  (± 0.54) |
|  |  | BC |  | -17.52  (± 0.40) | 12.41  (± 0.27) | 37.94  (± 1.73) | 10.00  (± 0.94) |
| 38 | Pycnogonid | HC |  | -19.54  (± 0.42) | 10.89  (± 0.31) | 40.37  (± 1.63) | 10.05  (± 0.63) |
|  |  | BC |  | -19.99  (± 0.48) | 10.75  (± 0.39) | 31.62  (± 9.56) | 8.15  (± 2.19) |
| 39 | Sabinea Shrimp | HC |  | -17.66  (± 0.24) | 13.50  (± 0.44) | 45.34  (± 0.39) | 13.35  (± 0.24) |
|  |  | NDC |  | -17.28  (± 0.21) | 13.26  (± 0.10) | 44.40  (± 1.35) | 13.64  (± 0.61) |
|  |  | BC |  | -16.98  (± 0.05) | 13.55  (± 0.20) | 44.59  (± 0.53) | 13.84  (± 0.21) |
| 40 | Sea Anemone | HC |  | -20.45  (± 0.27) | 10.86  (± 0.31) | 39.03  (± 1.84) | 9.88  (± 0.50) |
|  |  | NDC |  | -19.11  (± 0.34) | 11.93  (± 0.41) | 34.79  (± 2.87) | 9.89  (± 0.73) |
|  |  | BC |  | -19.33  (± 1.26) | 8.04  (± 2.50) | 35.89  (± 4.22) | 6.59  (± 2.02) |
| 41 | Snow Crab | HC |  | -17.70  (± 0.66) | 11.34  (± 0.28) | 37.24  (± 2.84) | 10.29  (± 1.21) |
|  |  | NDC |  | -15.33  (± 0.81) | 10.12  (± 0.68) | 30.71  (± 4.43) | 7.49  (± 1.76) |
|  |  | BC |  | -16.50  (± 0.79) | 10.71  (± 0.46) | 39.91  (± 7.16) | 10.58  (± 2.36) |
| 42 | Sea Star | HC |  | -12.90  (± 0.72) | 11.43  (± 1.39) | 21.10  (± 1.82) | 3.09  (± 0.60) |
|  |  | NDC |  | -12.54  (± 0.17) | 9.76  (± 0.36) | 19.04  (± 0.31) | 2.70  (± 0.14) |
|  |  | BC |  | -13.36  (± 0.42) | 12.09  (± 1.02) | 22.43  (± 2.09) | 3.81  (± 0.58) |
| 43 | Pandalus Shrimp | HC |  | -19.45  (± 0.07) | 10.69  (± 0.10) | 45.01  (± 0.53) | 13.78  (± 0.16) |
|  |  | NDC |  | -19.76  (± 0.10) | 10.58  (± 0.13) | 43.41  (± 0.30) | 12.95  (± 0.11) |
|  |  | BC |  | -18.88  (± 0.10) | 11.56  (± 0.23) | 48.06  (± 1.51) | 14.56  (± 0.36) |
| 44 | Sipunculid | HC |  | -19.09  (± 0.58) | 12.33  (± 0.93) | 13.45  (± 2.81) | 3.11  (± 0.55) |
|  |  | BC |  | -20.64  (± 0.20) | 8.79  (± 0.15) | 5.74  (± 0.54) | 1.30  (± 0.16) |
| 45 | Squid | HC |  | -19.86  (± 0.80) | 11.27  (± 0.22) | 43.82  (± 0.95) | 12.24  (± 0.07) |
|  |  | NDC |  | -21.12  (± 0.33) | 11.26  (± 0.53) | 46.78  (± 0.75) | 12.42  (± 0.25) |
|  |  | BC |  | -21.01  (± 0.22) | 11.33  (± 0.24) | 47.78  (± 0.63) | 10.98  (± 1.45) |
| 46 | Toad Crab | HC |  | -15.25  (± 0.31) | 9.64  (± 0.68) | 22.79  (± 0.67) | 4.47  (± 0.22) |
|  |  | NDC |  | -17.14  (± 1.80) | 9.85  (± 0.45) | 25.35  (± 2.10) | 5.07  (± 0.44) |
|  |  | BC |  | -15.92  (± 0.49) | 9.52  (± 0.10) | 26.05  (± 1.32) | 5.24  (± 0.50) |
| 47 | Isopod | HC |  | -18.36  (± 0) | 14.83  (± 0) | 54.07  (± 0) | 11.07  (± 0) |
| 48 | Nudibranch | HC |  | -16.60  (± 0) | 6.74  (± 0) | 31.57  (± 0) | 6.74  (± 0) |
| 49 | Sea Urchin | HC |  | -18.26  (± 1.26) | 7.97  (± 0.74) | 26.66  (± 6.61) | 4.71  (± 1.74) |
| 50 | Ostracod | NDC |  | -17.23  (±0) | 6.93  (±0) | 53.73  (± 0) | 5.34  (± 0) |
| 51 | Benthic Plants | HC |  | -17.86  (± 1.22) | 3.55  (± 1.44) | 33.37  (± 3.55) | 1.93  (± 0.15) |
|  |  | NDC |  | -18.41  (± 1.18) | 4.10  (± 1.23) | 35.37  (± 1.54) | 3.53  (± 0.72) |
|  |  | BC |  | -17.30  (± 0.16) | 5.19  (± 0.57) | 38.37  (± 0.04) | 2.82  (± 0.83) |
| 52 | Pelagic Algae | HC |  | -23.067  (± 0.903) | 6.143  (± 0.511) | 19.71  (± 4.45) | 4.33  (± 1.21) |
|  |  | NDC |  | -24.404  (± 0.683) | 5.211  (± 0.709) | 12.60  (± 1.30) | 2.40  (± 0.26) |
|  |  | BC |  | -21.98  (± 0.48) | 7.04  (± 1.04) | 30.37  (± 1.89) | 7.22  (± 0.74) |
