# Supplementary material for: Spatial variation in food web structure in a recovering marine ecosystem
Source: PLoS One. 2022 May 20;17(5):e0268440. doi: 10.1371/journal.pone.0268440 (PMC9122200; doi:10.1371/journal.pone.0268440)
Supplement: S5 Table — (DOCX) [file pone.0268440.s005.docx]

**S5** **Table**

|  | **Region** | **Small** | **Medium** | **Large** | **Stomach Samples** |
| --- | --- | --- | --- | --- | --- |
| **American Plaice** | HC | 4 | 10 | 7 | 26 |
|  | NDC | 5 | 12 | 4 | 2 |
|  | BC | 7 | 7 | 7 | 13 |
| **Atlantic Cod** | HC | 7 | 8 | 6 | 8 |
|  | NDC | 7 | 9 | 4 | 15 |
|  | BC | 7 | 7 | 7 | 16 |
| **Atlantic Herring** | HC | 0 | 2 | 0 | 0 |
|  | NDC | 4 | 3 | 1 | 18 |
|  | BC | 1 | 3 | 5 | 4 |
| **Capelin** | HC | 0 | 3 | 5 | 6 |
|  | NDC | 0 | 5 | 4 | 31 |
|  | BC | 3 | 3 | 3 | 14 |
| **Checker Eelpout** | HC | 3 | 3 | 3 | 9 |
|  | NDC | 0 | 0 | 0 | 0 |
|  | BC | 3 | 4 | 3 | 10 |
| **Greenland Halibut** | HC | 8 | 7 | 6 | 19 |
|  | NDC | 8 | 7 | 1 | 15 |
|  | BC | 5 | 9 | 7 | 15 |
| **Lanternfish** | HC | 3 | 3 | 4 | 30 |
|  | NDC | 3 | 4 | 2 | 8 |
|  | BC | 3 | 3 | 3 | 12 |
| **Redfish** | HC | 5 | 8 | 8 | 16 |
|  | NDC | 7 | 7 | 7 | 14 |
|  | BC | 7 | 7 | 7 | 7 |
| **Thorny Skate** | HC | 8 | 9 | 1 | 12 |
|  | NDC | 0 | 3 | 0 | 0 |
|  | BC | 7 | 11 | 3 | 26 |
