# Supplementary figures and images for: Spatial variation in food web structure in a recovering marine ecosystem
Source: PLoS One. 2022 May 20;17(5):e0268440. doi: 10.1371/journal.pone.0268440 (PMC9122200; doi:10.1371/journal.pone.0268440)

**BC**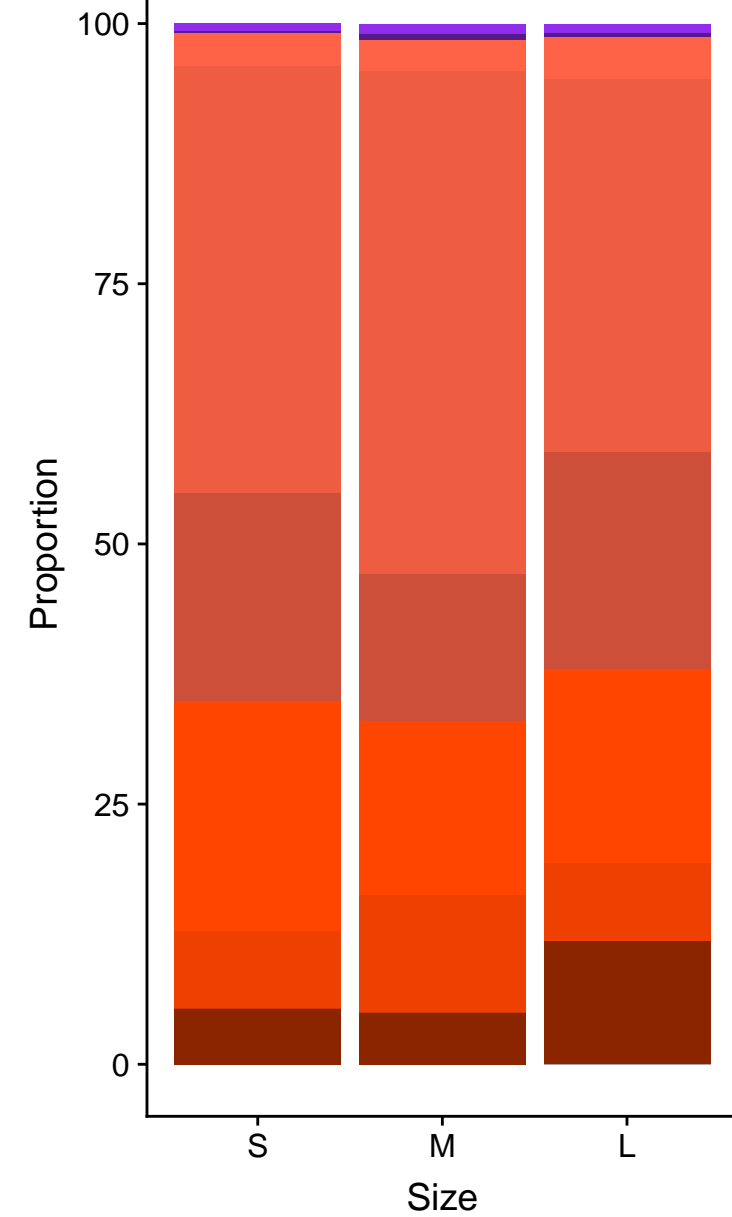**NDC**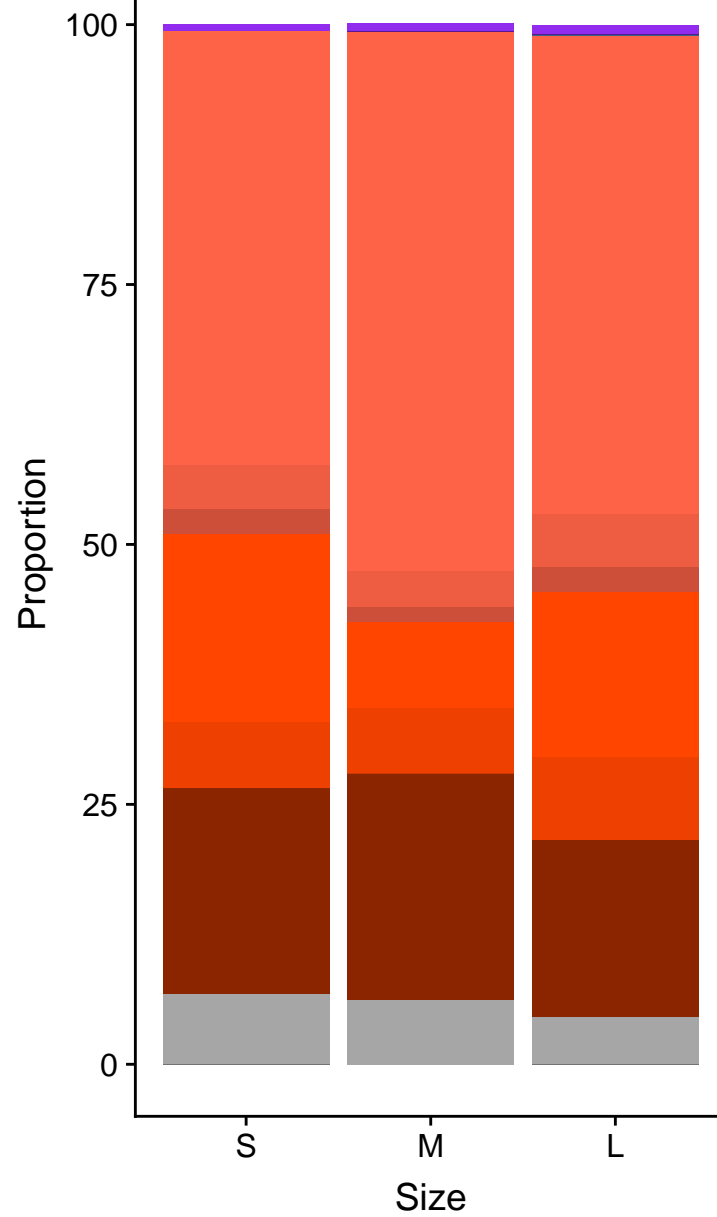**HC**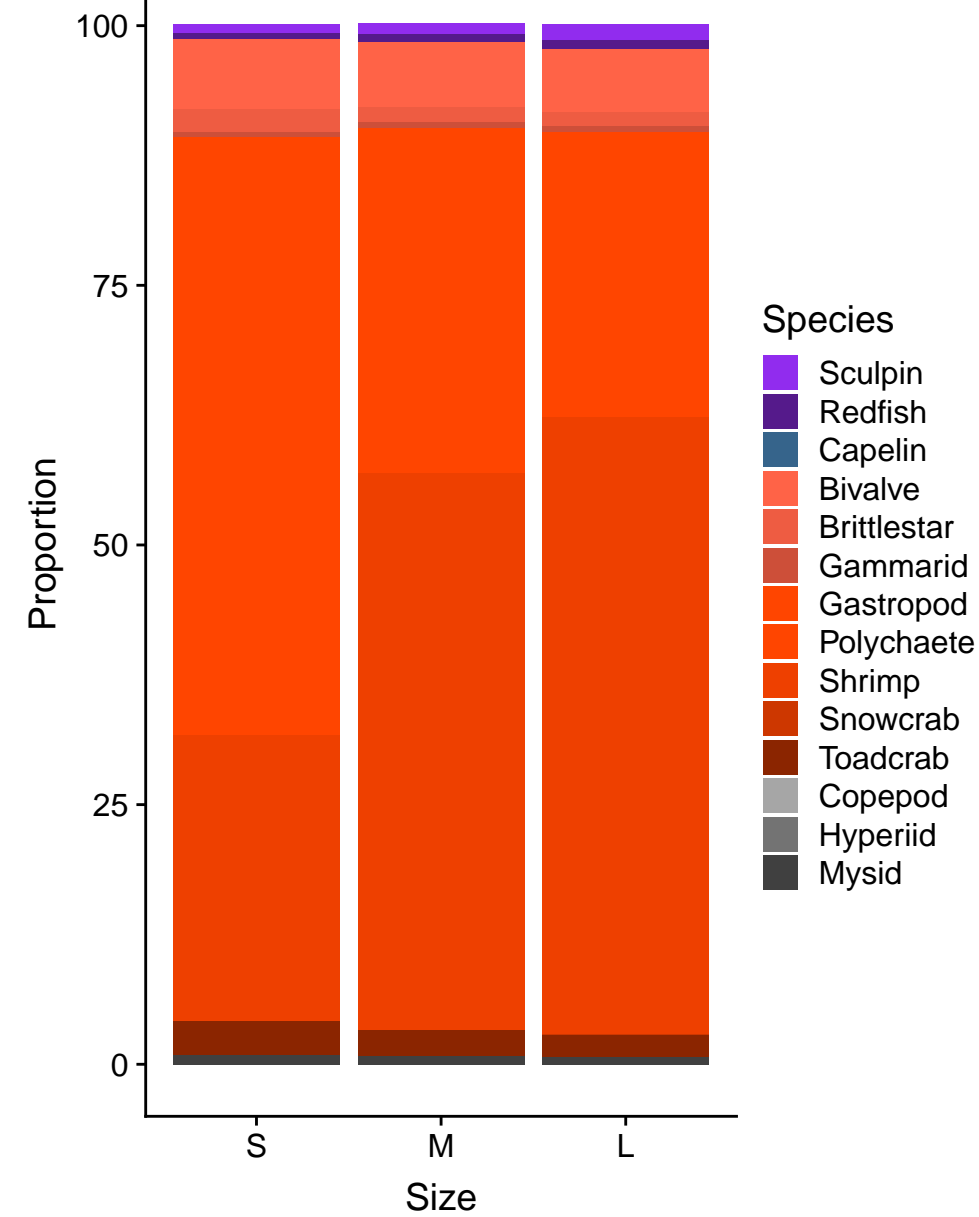

Supplement: S1 Fig — (PDF) [file pone.0268440.s006.pdf]

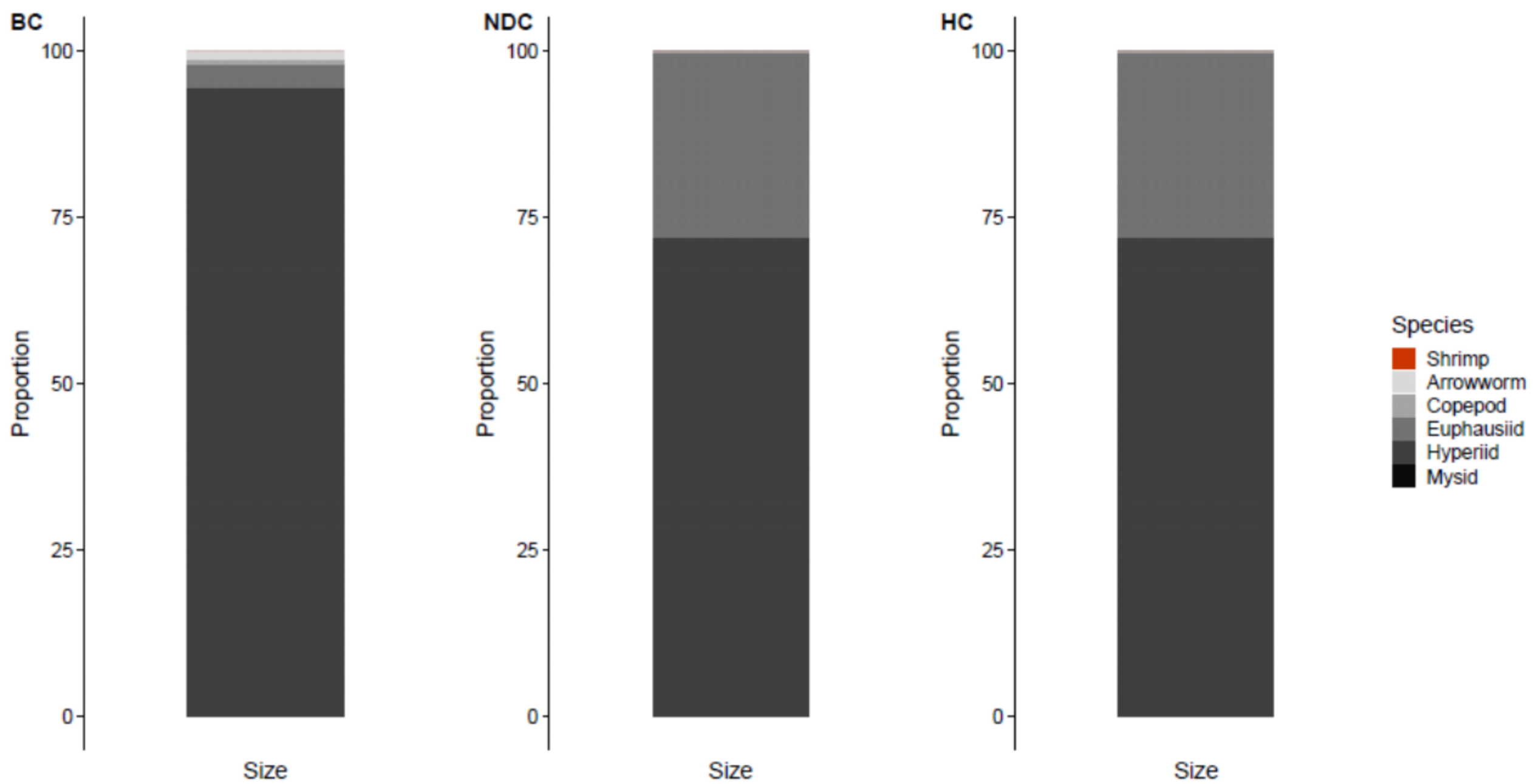

Supplement: S2 Fig — (PDF) [file pone.0268440.s007.pdf]

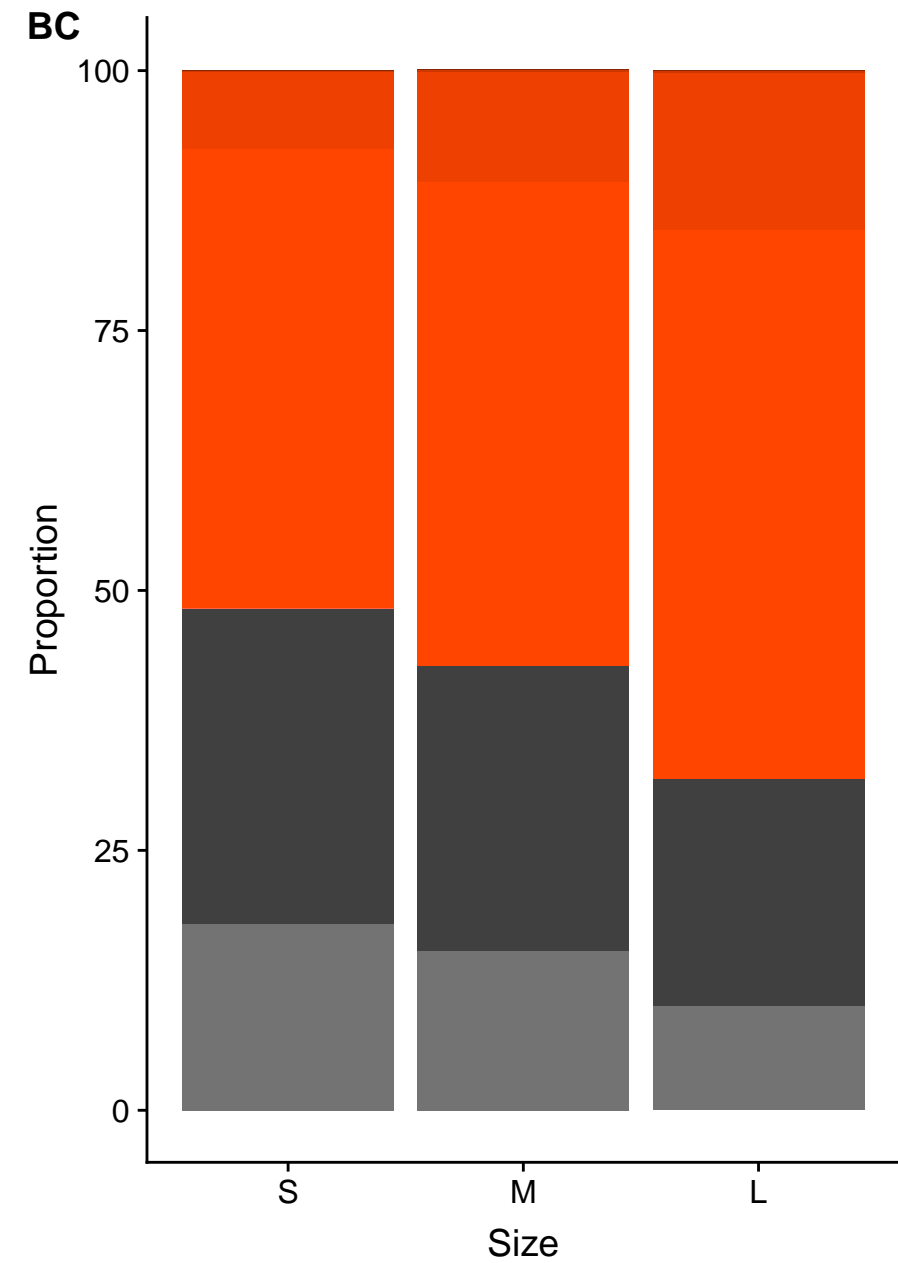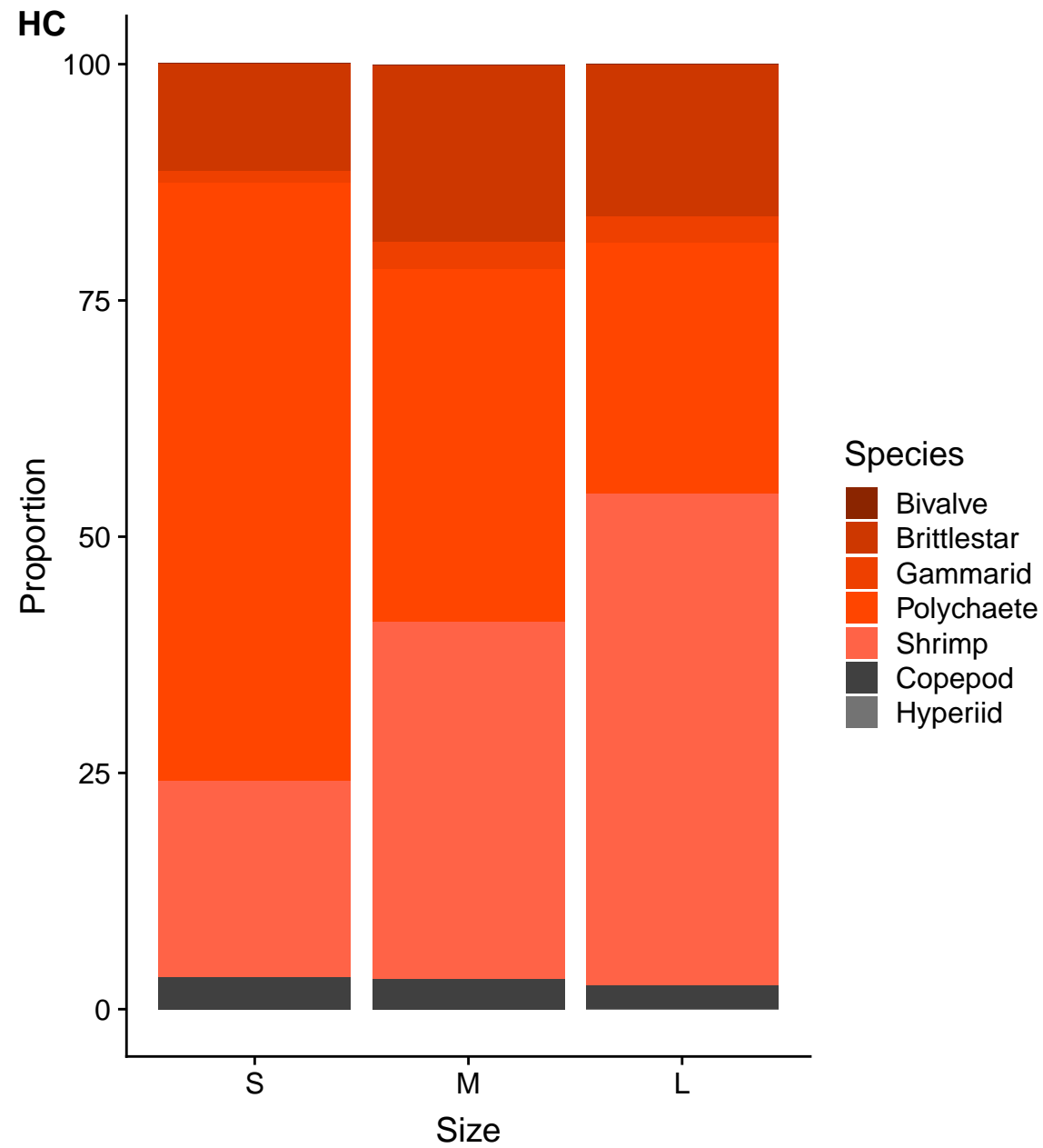

Supplement: S4 Fig — (PDF) [file pone.0268440.s009.pdf]
