# Supplementary material for: Spatial variation in food web structure in a recovering marine ecosystem
Source: PLoS One. 2022 May 20;17(5):e0268440. doi: 10.1371/journal.pone.0268440 (PMC9122200; doi:10.1371/journal.pone.0268440)
Supplement: S3 Fig — (PDF) [file pone.0268440.s008.pdf]

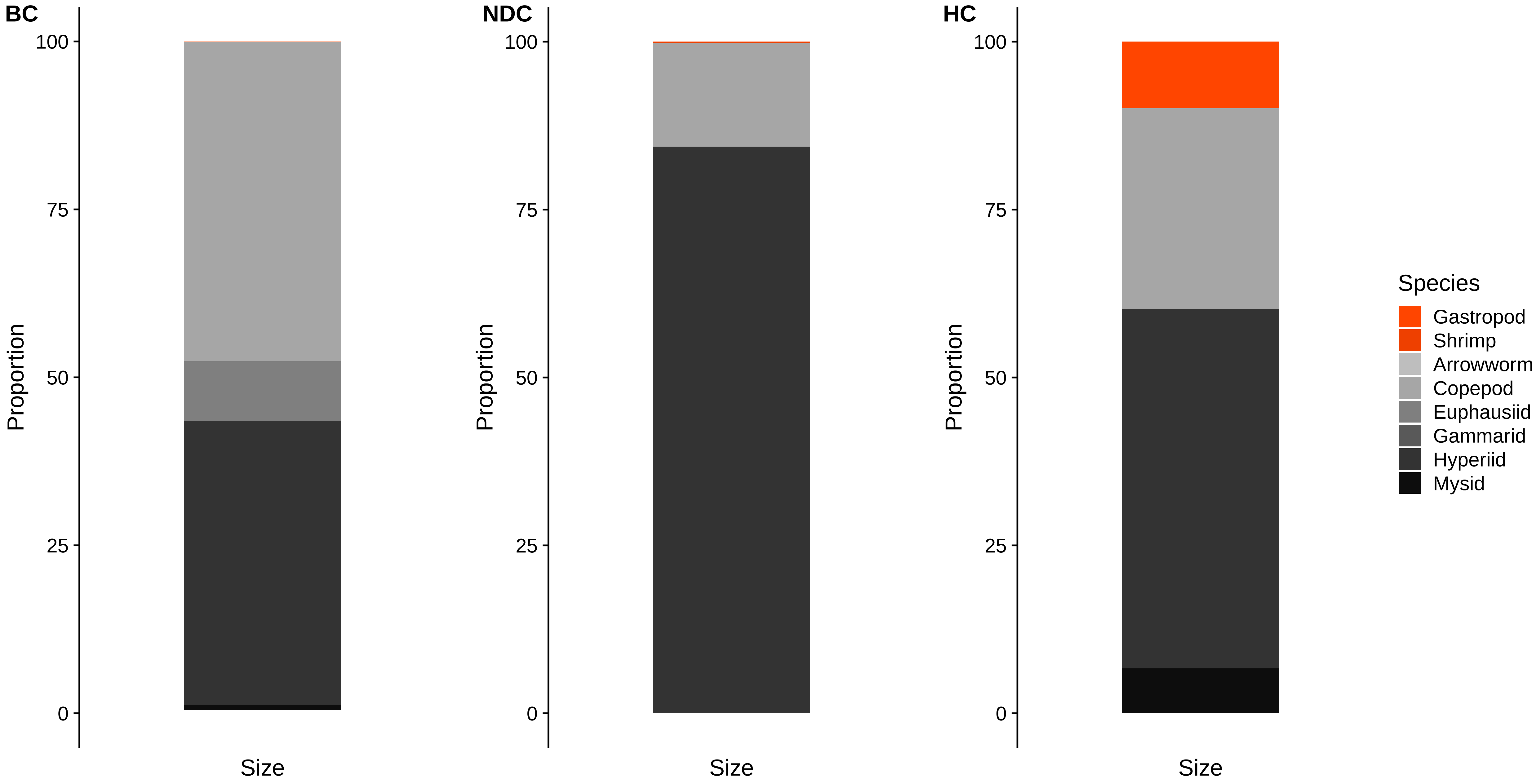

BC
NDC
HC
Proportion
100
75
50
25
0
Size
Species
Gastropod
Shrimp
Arrowworm
Copepod
Euphausiid
Gammarid
Hyperiid
Mysid
