# Supplementary material for: Spatial variation in food web structure in a recovering marine ecosystem
Source: PLoS One. 2022 May 20;17(5):e0268440. doi: 10.1371/journal.pone.0268440 (PMC9122200; doi:10.1371/journal.pone.0268440)
Supplement: S6 Fig — Functional group categories are designated by two letter abbreviations: PI for pelagic invertebrates, BI for benthic invertebrates, PF for pelagic fish, DF for demersal fish. (PDF) [file pone.0268440.s011.pdf]

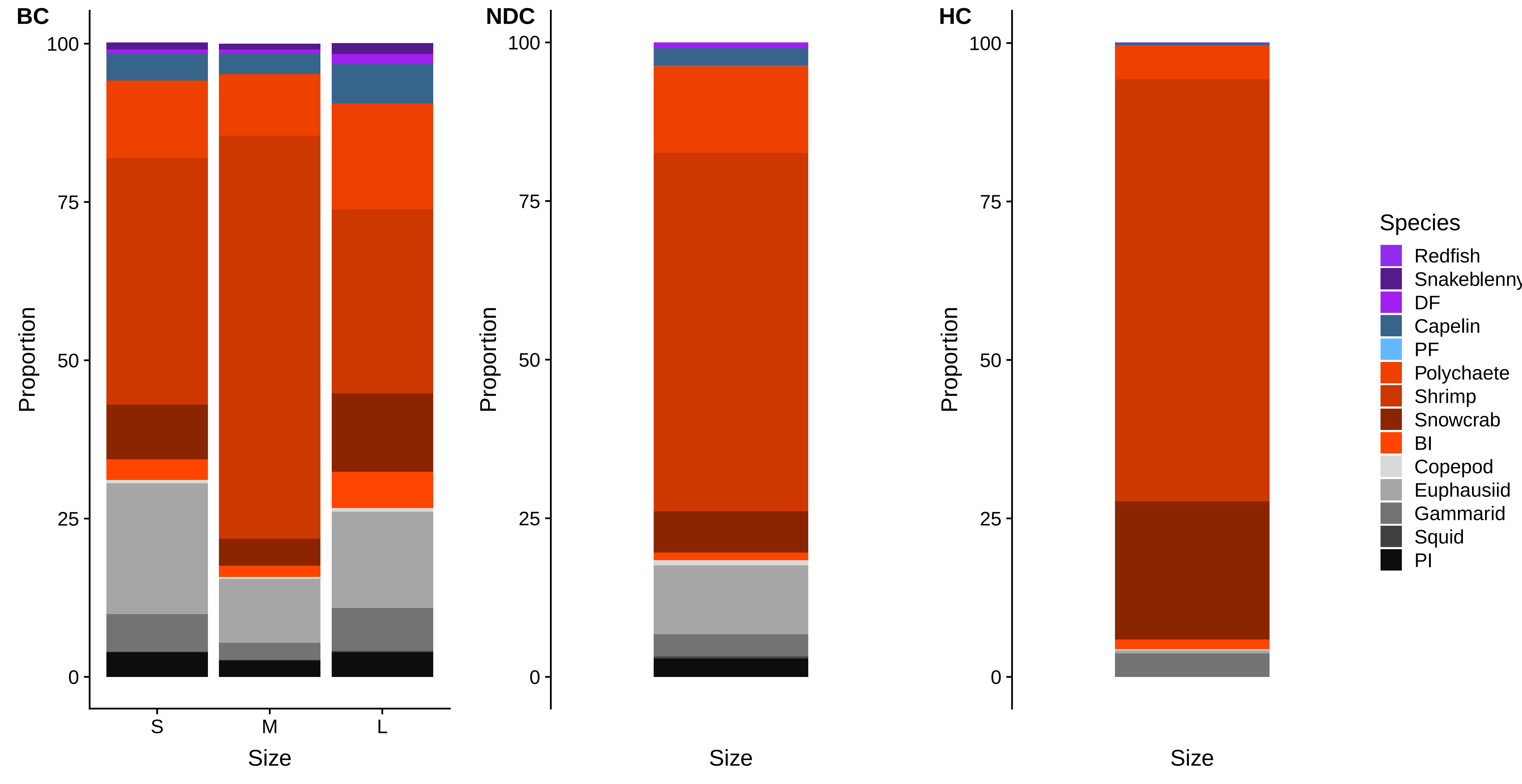
BC
NDC
HC
100
75
50
25
0
Proportion
S
M
L
Size
Species
Redfish
Snakeblenny
DF
Capelin
PF
Polychaete
Shrimp
Snowcrab
BI
Copepod
Euphausiid
Gammarid
Squid
PI
